# Developmental and Genotypic Variation in Leaf Wax Content and Composition, and in Expression of Wax Biosynthetic Genes in *Brassica oleracea* var. *capitata*

**DOI:** 10.3389/fpls.2016.01972

**Published:** 2017-01-09

**Authors:** Rawnak Laila, Arif Hasan Khan Robin, Kiwoung Yang, Jong-In Park, Mi Chung Suh, Juyoung Kim, Ill-Sup Nou

**Affiliations:** ^1^Department of Horticulture, Sunchon National UniversitySuncheon, South Korea; ^2^Department of Bioenergy Science and Technology, Chonnam National UniversityGwangju, South Korea

**Keywords:** wax biosynthetic genes, wax composition, wax formation, wax crystals, expression analysis, *Brassica oleracea* var. *capitata*

## Abstract

Cuticular waxes act as a protective barrier against environmental stresses. In the present study, we investigated developmental and genotypic variation in wax formation of cabbage lines, with a view to understand the related morphology, genetics and biochemistry. Our studies revealed that the relative expression levels of wax biosynthetic genes in the first-formed leaf of the highest-wax line remained constantly higher but were decreased in other genotypes with leaf aging. Similarly, the expression of most of the tested genes exhibited decrease from the inner leaves to the outer leaves of 5-month-old cabbage heads in the low-wax lines in contrast to the highest-wax line. In 10-week-old plants, expression of wax biosynthetic genes followed a quadratic function and was generally increased in the early developing leaves but substantially decreased at the older leaves. The waxy compounds in all cabbage lines were predominately C_29_-alkane, -secondary alcohol, and -ketone. Its deposition was increased with leaf age in 5-month-old plants. The high-wax lines had dense, prominent and larger crystals on the leaf surface compared to low-wax lines under scanning electron microscopy. Principal component analysis revealed that the higher expression of *LTP2* genes in the lowest-wax line and the higher expression of *CER3* gene in the highest-wax line were probably associated with the comparatively lower and higher wax content in those two lines, respectively. This study furthers our understanding of the relationships between the expression of wax biosynthetic genes and the wax deposition in cabbage lines.

**Highlight:** In cabbage, expression of wax-biosynthetic genes was generally decreased in older and senescing leaves, while wax deposition was increased with leaf aging, and C_29_-hydrocarbon was predominant in the wax crystals.

## Introduction

Plants are exposed to a broad range of biotic and abiotic stresses including drought, cold, ultraviolet (UV) light and pathogen attack during their growth and development. The cuticle present in leaf surfaces acts as a defensive barrier against pests and pathogens and provides protection from UV radiation (Wink, 1988; Holmes and Keiller, 2002; Bargel et al., 2004; Yeats and Rose, 2013). The cuticle comprises two different types of biochemical compounds including a lipophilic cutin polymer matrix and waxes (Holloway, 1982; Jeffree, 1996; Kunst et al., 2005). In addition to defensive roles, the cuticular waxes at the substomatal chambers of the abaxial surfaces of leaves help prevent uncontrolled evaporation of water. Cuticular wax is arranged in distinct layers thought to have different chemical compositions (Jetter et al., 2000; Knoche et al., 2000). The cuticular wax layer consists of very long-chain hydrocarbon compounds (VLCHCs; C20 to C34). These hydrocarbon compounds include alkanes, primary alcohols, aldehydes, secondary alcohols, ketones, esters and often triterpenoids and flavonoids as derived compounds (Jetter et al., 2008; Samuels et al., 2008). Genetic and environmental factors are largely responsible for the variable composition of wax (Bianchi, 1995; von Wettstein-Knowles, 1995; Post-Beittenmiller, 1996). A number of gene loci are reported to be involved in wax biosynthesis of plants; these genes regulate transcription, mRNA stability and post-translational modification in waxy and wax-less plants (von Wettstein-Knowles, 1995; Lee and Suh, 2013; Pu et al., 2013; Lee et al., 2015). However, the genetic mechanism involved in accumulation of low- and high-wax in plants remains a subject for further investigation. Wax biosynthesis occurs via two distinct biosynthetic pathways, (i) the acyl reduction pathway and (ii) the decarbonylation pathway. The acyl-reduction pathway involves modification of VLCHCs into aldehydes and primary alcohols. The resulting primary alcohols may be esterified to form wax esters. The decarbonylation pathway produces aldehydes, alkanes, secondary alcohols, and ketones (Kunst and Samuels, 2003; Samuels et al., 2008).

**FIGURE 1:**
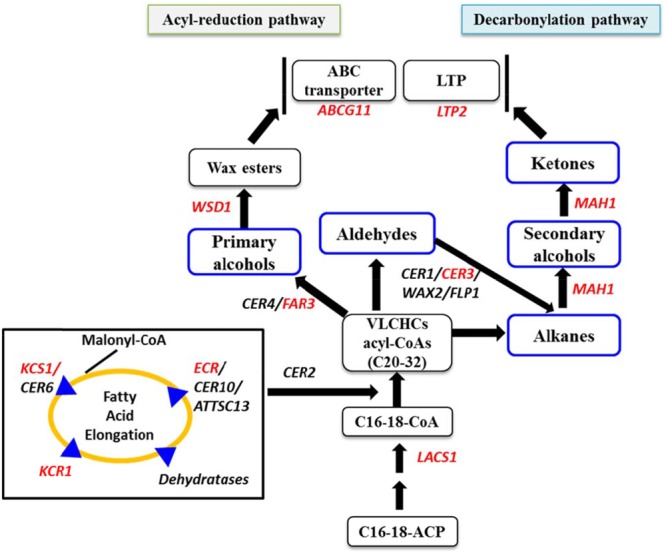
**Simplified cuticular wax biosynthetic pathway adapted from Kunst and Samuels (2009), Yeats and Rose (2013), and Lee et al. (2015).** Red letters indicate the genes studied by qPCR for relative expression level. Blue boxes indicate biochemical composition of cuticular waxes. LACS1, long chain acyl-CoA synthase 1; KCS1, 3-ketoacyl-CoA synthase; ECR, enoyl-CoA reductase; CER3, protein ECERIFERUM 3; MAH1, midchain alkane hydroxylase 1; CER4 encodes an alcohol-forming fatty acyl-coenzyme A reductase (FAR); LTP2, lipid transfer proteins; FAR3, fatty acyl-CoA reductase; WSD1, wax ester synthase/acyl-coenzyme A: diacylglycerol acyltransferase; ABCG11, ATP-binding cassette (ABC) transporter; C16-18-ACP, C16-C18-Acyl carrier protein; C16-18-CoA, C16-18-Coenzyme-A. LACS1 participates in converting free fatty acids into CoA thioesters.

A number of proteins and coenzymes are involved in the acyl reduction and decarbonylation pathways. For example, long chain acyl-Coenzyme A (CoA) synthetase 1 (LACS1) participates in converting free fatty acids into CoA thioesters, which are precursors for VLCHCs of both the acyl reduction and the decarbonylation pathways (**Figure 1**; Lü et al., 2009; Weng et al., 2010; Jessen et al., 2011). Four sequential enzymatic reactions are involved in chain elongation of hydrocarbons resulting in VLCHCs, (i) condensation of two-carbon units to acyl-CoA by 3-ketoacyl-CoA synthase (KCS), (ii) reduction of 3-ketoacyl-CoA by 3-ketoacyl-CoA reductase (KCR), (iii) dehydration of 3-hydroxyacyl-CoA by 3-hydroxyacyl-CoA dehydratase, and (iv) reduction of *trans*-2,3-enoyl-CoA by *trans*-2-enoyl-CoA reductase (ECR) (**Figure 1**; Todd et al., 1999; Fiebig et al., 2000; Zheng et al., 2005; Bach et al., 2008; Beaudoin et al., 2009). In the acyl-reduction pathway, a primary alcohol is produced from the VLCHCs by fatty acyl-CoA reductase (FAR3/CER4) and may then be converted into wax esters by WSD1 (**Figure 1**). The wax biosynthesis process begins in the endoplasmic reticulum with the production of cuticular wax components, which are transported to the plasma membrane, and then exported to the apoplast via an ATP-binding cassette (ABC) transporter (McFarlane et al., 2010). By contrast, in the decarbonylation pathway, midchain alkane hydroxylase 1 (MAH1) converts an alkane, produced from the VLCHCs, into a secondary alcohol and subsequently oxidizes the secondary alcohol to a ketone (**Figure 1**). A multiprotein complex consisting of CER3 and CER1 helps catalyze the conversion of VLC acyl-CoA to the corresponding VLC alkane (**Figure 1**; Bernard and Joubès, 2013). Lipid transfer proteins are fundamental proteins capable of transferring lipids between natural and artificial membranes *in vitro* and are thought to be involved in transporting cuticular wax components through the hydrophilic cell wall (Kader, 1996; DeBono et al., 2009; Kim et al., 2012). For example, GPI-anchored lipid transfer protein 1 (LTPG/LTPG1) and LTPG2 are directly or indirectly involved in the export or deposition of cuticular waxes in the cell walls of expanding epidermal cells and certain secretary tissues (Kader, 1996; DeBono et al., 2009; Lee et al., 2009a; Kim et al., 2012).

Although, the biochemical processes involved in two wax biosynthetic process are quite well-understood, the genetics of wax biosynthesis is much less studied in the Brassicaceae family. The epicuticular wax composition influences leaf color, plant–insect interactions, and plant development in the Brassicaceae (Eigenbrode, 1996; Eigenbrode and Kabalo, 1999; Picoaga et al., 2003). In *Arabidopsis*, cuticle composition and gene expression analyses indicate that wax biosynthesis is induced by water deficit, sodium chloride, and abscisic acid (ABA) treatments (Kosma et al., 2009). The quality and quantity of plant cuticular waxes widely vary by species and type of organ and additionally respond to environmental growth conditions (Riederer and Muller, 2008). As wax deposition is associated with abiotic and biotic stress tolerance, it has become increasingly important to understand the detailed genetic behavior of wax biosynthetic genes.

Cabbage, *Brassica oleracea* var. *capitata*, is an important vegetable subspecies that can suffer from various kinds of biotic and abiotic stresses due to the absence of biochemical and morphological defense mechanisms in different genotypes. Some genotypes produce cuticular waxes in abundance for protection from biotic and abiotic stresses. Understanding the ultrastructural morphology, genetics and biochemistry of wax biosynthesis in cabbage plants is important to develop cabbage inbred lines with inherent resistant capacities against different stresses. A notable number of studies have investigated the wax composition in *B. oleracea* and *Arabidopsis thaliana* (Jenks et al., 1995; Bernard and Joubès, 2013; Lee et al., 2015). In broccoli, C_29_ -alkane, -ketone, and -secondary alcohol were the dominant wax compounds (Lee et al., 2015). However, data are scarce regarding ultra-structural morphology of cabbage leaf waxes and the expression patterns of associated genes in *B. oleracea* var. *capitata*. A few studies in broccoli have reported the identification of a lipid transfer protein related to wax biosynthesis (Pyee et al., 1994; Pyee and Kolattukudy, 1995), the biochemical composition of wax compounds, and the expression patterns of various wax biosynthetic genes (Lee et al., 2015). However, the wax formation pattern in cabbage has not yet been studied in detail, and little is known about the expression patterns of genes related to wax biosynthesis in cabbage leaves at different developmental stages. Expression levels of wax biosynthetic genes involved in different steps of wax formation likely reflect the wax formation behavior of cabbage at different developmental stages. It is important to understand expression patterns of wax biosynthetic genes under optimal growth condition for genetically breeding the expected wax content. Relevant data might also provide an indication of how the cuticular wax components and crystal structure in leaves change with increasing plant age. To date, the optimum level of cuticular wax that a cabbage genotype could be bred for has not been modeled, although the cuticular waxes have vital defensive roles for the plants against various environmental stresses.

In this study, we observed the deposition and distribution patterns of wax compounds on the cabbage leaf surface using scanning electron microscopy. We also quantified and compared cuticular wax components in four cabbage inbred lines of contrasting phenotypes. Finally, we identified genes related to wax biosynthesis and examined their expression patterns in the edible organs of cabbage inbred lines at three different developmental stages. The results of this analysis help elucidate the wax formation patterns in different cabbage genotypes and their association with the related genes.

## Materials and Methods

### Plant Materials and Growth Conditions

Seeds of two different groups of *B. oleracea*, two genotypes from each group, were obtained from Asia Seed, Co., Ltd (Seoul, South Korea). The groups included high-wax lines (BN4383, BN4384) and low-wax lines (BN4071, BN4161) (**Table 1**; Supplementary Figure S1). The seedlings were grown in soil-based compost under standard growth chamber conditions. Three separate experiments were conducted as presented in **Table 1**. Leaf samples from 2- to 4-week-old plants were collected for Experiment 1 to investigate the variation in expression of wax biosynthesis-related genes at the seedling stage (**Table 1**; Supplementary Figure S1). Another set of plants were allowed to grow 10 weeks and then leaf samples were destructively harvested as a part of Experiment 2 (**Table 1**; Supplementary Figure S2). Biochemical composition of wax compounds was also studied for the leaf samples collected from the leaf at position 4 considering the youngest leaf as a reference point in Experiment 2 (Supplementary Figure S2). Leaf appearance intervals in all four lines were recorded in this experiment. In Experiment 3, to study the expression level of genes and to investigate the ultra-structure of wax crystals from the inner, middle and outer positions of 5-month-old cabbage heads, greenhouse-grown plants were collected from the Asia Seed, Co., Ltd (Seoul, South Korea) (**Table 1**; Supplementary Figure S1). In Experiment 3, the low-wax line BN4161 was replaced by another low-wax line, BN4083, as BN4161 was unavailable (**Table 1**; Supplementary Figure S1). In addition to expression profiling of the leaves of inner, middle, and outer positions, the deposition of wax was investigated at these three leaf positions under a scanning electron microscope (**Table 1**; Supplementary Figure S1). All collected samples to be used for expression profiling of wax biosynthesis genes were immediately frozen in liquid nitrogen, and stored at -80°C until RNA isolation and cDNA synthesis. Length and diameter of the individual leaves from all three experiments were recorded and presented in Supplementary Table S1.

**Table 1:** Cabbage (*Brassica oleracea* var. *capitata*) lines used to study the expression of wax biosynthesis-related genes and to estimate cuticular wax and epicuticular wax crystallization patterns.

Experiment	Objectives	Sampling time points	Developmental status of leaves	Results presented in	Genotype
1	Determine effect of leaf age on wax biosynthesis gene expression	The first-formed true leaf was collected at week 2, week 3, and week 4 after transplantation	The first-formed leaf was developing from week 2 to week 4	**Figure 4**	BN4383BN4384BN4071BN4161^∗^
2	(i) Same as Experiment 1, (ii) determine variation in wax biochemical compounds among genotypes	Leaves at the following positions: 1, 2, 3, 4, 8, and 12 of approximately known age at the stem axis. Samples were destructively harvested at a single time point from the 10-week-old plants	The leaves were expanding from leaf position 1 to leaf position 5. Leaves were nearly fully expanded at leaf position 4	**Figures 3, 5**, and **7**; **Table 3**	
3	(i) Same as Experiment 1(ii) Determine variation in wax deposition at the leaf surface	Outer, middle and inner leaves as in **(Figure 2)**. Plants were destructively harvested at 5 months of age	Inner and middle leaves were expanding. Outer leaf was fully expanded	**Figures 2** and **6**	BN4383BN4384BN4071BN4083^∗^

*The lines BN4383 and BN4384 were high-wax depositing and the other lines were low-wax depositing.*

*^∗^In Experiment 3 the low-wax depositing line BN4161 was replaced by BN4083.*

### Scanning Electron Microscopy (SEM)

Leaf samples collected from inner, middle, and outer leaf positions of 5-month-old plants were cut into about 1 cm^2^ pieces and briefly treated with frozen methanol in liquid nitrogen (Pathan et al., 2010). The frozen samples were freeze-dried for 24 h prior to examination of epicuticular wax crystallization patterns. Each leaf sample was extensively scanned under a scanning electron microscope at four different magnifications: 1000×, 2000×, 5000×, and 10000×. Images were captured only at the most dense waxy regions of the leaf segments. Freeze-dried samples were prepared and viewed by a cryo-SEM as described by Lü et al. (2009).

### Extraction of Cuticular Wax and GC–MS Analysis

Fresh leaves of leaf position 4 from 10-week-old plants were subjected to gas chromatography–mass spectrometry (GC–MS) analysis. Cuticular wax was extracted from the three selected biological replicates and each biological replicate was analyzed three times by GC–MS as described by Lee et al. (2009a,b) and Kim et al. (2012). Approximately 0.2 g of fresh cabbage leaves was sampled using a cork borer of known diameter (Bokel Scientific, 12 size). The samples were then treated with 5 mL chloroform (CHCl_3_) for 15 s at room temperature. The chloroform extract was supplemented with *N-octacosane* (1 mg g^-1^ fresh weight), docosanoic acid (50 ug g^-1^ fresh weight), and 1-tricosanol (100 ug g^-1^ fresh weight) as internal standards. The samples were then heated at 40°C under a gentle stream of nitrogen gas to remove the solvent. The solvent-removed wax mixtures were then dissolved in a mixture of 100 μL bis-*N*,*N*-(trimethylsilyl)trifluoroacetamide (Sigma) and 100 μL pyridine. Each sample was heated at 90°C for 30 min to exchange all hydroxyl-containing compounds for the corresponding trimethylsilyl (TMSi) derivatives. The samples were then evaporated to dryness with nitrogen followed by dissolving in a 1:1 (v/v) solution of heptane: toluene. A DB-5MS (length 30 mm, inner diameter 0.25 mm, film thickness 0.25 μm, Agilent) column and a DB-5 (length 30 mm, inner diameter 0.25 mm, film thickness 0.25 μm, Agilent) column were used for GC–MS and GC analysis, respectively. The qualitative composition was evaluated by a capillary GC–MS (GCMS-QP2010, Shimadzu, Japan). During this process a He carrier gas inlet flow rate of 1.0 mL min^-1^ (column flow rate of 0.74 mL min^-1^) and a mass spectrometric detector (GCMS-QP2010, Shimadzu) were used. The GC–MS procedure was conducted at a high temperature ranging between 220 and 300°C. This protocol involves injection at 220°C, maintenance of the temperature at 220°C for 4.5 min followed by an increase of the temperature up to 300°C at a rate of 3°C min^-1^. The subsequent steps took around 30 min: maintaining 300°C for 10 min, raising the temperature up to 320°C at a rate of 2°C min^-1^ and maintaining that temperature for another 10 min. For MS conditions, ion source and interface temperature were 230 and 250°C, respectively, and detector voltage was 1.2 kV (absolute) with 0.5 s scan interval; the m/z value range was from 50 to 600. The mixtures were then subjected to quantitative analysis using a capillary GC (GC-2010, Shimadzu) with a flame ionization detector. Individual compounds were evaluated against the internal standard, with the peak areas integrated automatically. To quantify wax compounds, C_22_ fatty acid was the standard for VLCFAs and the unidentified, C_23_ primary alcohol was the standard for primary and secondary alcohols; C_28_ alkane was the standard for alkanes, ketones, ketols, and aldehydes (Supplementary Figure S3).

### Selection of Wax Biosynthetic Genes for Expression Analysis

A list of wax biosynthesis-related genes from *Arabidopsis* along with their accession numbers were obtained from Lee et al. (2015) (**Table 2**). The coding DNA sequence (CDS) of each accession was then obtained from NCBI^1^. The *B. oleracea* orthologs for each *Arabidopsis* wax biosynthetic gene was then obtained from the Bolbase database^2^ by similarity searches (**Table 2**; Supplementary Data File). The sub-cellular localization of the corresponding wax biosynthesis proteins was predicted using ProtComp 9.0 from Softberry^3^ (**Table 2**; Supplementary Data File). A phylogenetic tree was constructed to confirm that each of the reported *Arabidopsis thaliana* wax biosynthesis genes clustered with the selected *B. oleracea* genes of similar function (Supplementary Figure S4).

**Table 2:** List of primer sequences used for qRT-PCR of cuticular wax biosynthetic genes.

*Arabidopsis* homolog (accession number)	Gene name	Bol Id	cDNA size (bp)	Primer forward (F) and reverse (R)	Product size (bp)	Sub-cellular localization
NM _130292	*BoLACS1.1*	Bol002529	1983	F: TGAGCTTACTGATGAAGTCTTGR: ACAGAGTTTTGACCGTAGATGT	174	Endoplasmic reticulum
	*BoLACS1.3*	Bol029614	1755	F: CCATTCGAAGAACTATGCTCR: CATTTCGTCGACTTGTACCT	245	
	*BoLACS1.4*	Bol002590	1983	F: CATAAAACGTTGGGCTAAAGR: TACTTGAGCAGGTTGTTCCT	231	
NM_099994.3	*BoKCS1.1*	Bol018447	1563	F: AAGTCGGAATCTTGATCGTAR: ATTGCCGAAGTACCAGTTTA	239	Plasma membrane
	*BoKCS1.2*	Bol000521	738	F: AAATGTCTGTGGACTCGTTCR: CATATCTAGCTTCGGAGGTG	158	
	*BoKCS1.3*	Bol040715	1590	F: GCCATTATACGAATCCAGAGR: GCTCAGAGAGCATATCCAAC	184	
NM_105441.2	*BoKCR1.1*	Bol010474	636	F: GATCAATGTTGAGGGGACTAR: CCTTGAGAACTGATCCACAT	172	Endoplasmic reticulum
NM_115394.3	*BoECR*	Bol044348	933	F: TAGGGTTCAACATCGCTACTR: TCACCCATCTTCTTGGATAC	156	
NM_125164.2	*BoCER3.3*	Bol012187	1893	F: ATGGTAGCTTTTTCAGCTTGR: CTCCAAAGAACATGAAGGAA	182	Plasma membrane
	*BoCER3.4*	Bol015584	1893	F: GGAACTGCACTTATGGTGATR: ACCAGATCAATACGGTCAAC	187	
NM_001124037.1	*BoMAH1.1*	Bol016302	1491	F: CACGACAAGTTCAGTTCTCAR: AAGGAGACTTGTGGTTGAAG	191	
NM_129410.4	*BoLTP2.1*	Bol017820	405	F: AGCGGCGTTACTAGTCTAAAR: TGTAAGGAATGTTGACTCCA	154	Extracellular
	*BoLTP2.2*	Bol025301	360	F: GCTAGAGCCTTAGGCCCTAR: TGTTGCAGTTGGTATTGGTG	106	
	*BoLTP2.3*	Bol025304	360	F: GCTAGCGCCTTAGGCCCTAR: CTTGTTGCAGTTGGTGTTG	108	
NM_119537.6	*BoFAR3.1*	Bol013612	1452	F: GTTCAAGCTGGAAAACAGAAR: TTCTTTACACGAACCACCTT	152	Extracellular
	*BoFAR3.2*	Bol017561	1467	F: GTCCATATTGGTCATTGGAGR: CTTCTCTTTCACCACCTTGT	184	Chloroplast
NM_123089.2	*BoWSD1.2*	Bol020399	1443	F: TTGGGTTCCTGTTAATGTTCR: AGATTCTGCGTTTGATGTCT	187	Endoplasmic reticulum
	*BoWSD1.3*	Bol024738	519	F: AATCTCTTATGTCGCAGGAAR: AGCGCTTCTACAATTTCATC	153	
NM_101647.4	*BoABCG11.1*	Bol013247	2112	F: TCTTCATCCAGGATTCACTCR: GCCAGAAAAGTTTTGGTATG	205	Plasma membrane
	*BoABCG11.2*	Bol030816	2103	F: TGGAGAGAGAAAACACCAACR: GCGTCGAGCATAGTAGATTT	197	
AF044573	*actin1*		1500	F: TTCTCTCTTCCACACGCCATR: CTTGTCCTGCGGGTAATTCG	235	
JQ435879	*cctin2*		438	F: GTCGCTATTCAAGCTGTTCTCTR: GAGAGCTTCTCCTTGATGTCTC	251	
XM_013753106	*actin3*		1634	F: ATCACACTTTCTACAATGAGCR: TCGTAGATTGGCACAGTGTGAG	241	

**Primer to test gDNA contamination**					

		Bol010474	Intron2	F: TGCTTTGTTTTGCTGCGTCTR: AAGGCTATTGGGCAGCGTTA	509	

### cDNA Synthesis and qRT-PCR Expression Analysis

Total RNA was extracted with RNeasy Plant Mini Kit (Qiagen, USA) using 70 mg leaf samples after grinding with liquid nitrogen and treatment with RNase-free DNase (Qiagen, Hilden, Germany). RNA purity was determined by the 260/280 nm ratio, quantified with a Nanodrop^®^ ND-1000 (Thermo Scientific, Hudson, NH, USA) and integrity checked by electrophoresis. For cDNA synthesis, the Superscript^®^ III First-Strand synthesis kit (Invitrogen, Carlsbad, CA, USA) was used according to the manufacturer’s instructions. The cDNA was tested for gDNA contamination by RT-PCR using primers that would amplify only a genomic product, and the cDNA was found to be contamination free (**Table 2**). To determine the expression patterns of the wax biosynthetic genes, qRT-PCR was performed using gene-specific primers, which were designed using Primer 3^4^ and are listed in **Table 2**. Melting curve analysis was performed to test for primer specificity. Primer efficiency was tested for each primer by running a dilution series as described by Robin et al. (2016). Three different *B. oleracea actin* genes (GenBank Accession No. AF044573, JQ435879, and XM_013753106) were used as housekeeping genes (Robin et al., 2016; **Table 2**). Expression levels of the housekeeping gene were stable across different genotypes, leaf ages and organs of *B. oleracea* var. *capitata*. Real-time quantitative PCR was performed using 1 μL (containing 50 ng) cDNA in a 20-μL reaction employing qPCRBIO syGreen Mix Lo-ROX (PCR Biosystems, London, UK). The conditions for real-time PCR were as follows: 10 min at 95°C, followed by 40 cycles at 95°C for 20 s, 58°C for 20 s, and 72°C for 25 s. The fluorescence was recorded at the last step of each cycle. There were three biological replicates for each sample. Biological replicates were cultured under the same growing conditions. Each biological replicate was repeated three times. Detection of amplification and analysis of data were conducted using a LightCycler96 (Roche, Mannheim, Germany). Relative gene expression was calculated following the 2^-ΔΔCT^ method (Livak and Schmittgen, 2001). Leaf samples collected from the first-formed true leaf of 2-week-old plants in Experiment 1, the youngest leaf (Leaf 1) collected from 10-week-old plants in Experiment 2, and the inner leaf collected from 5-month-old plants in Experiment 3 were the calibrators for calculating the relative expression level of other leaf samples for each gene (Supplementary Figure S1). For analysis of genotypic variation in relative expression, inbred line BN4383 was used as the calibrator.

### Statistical Analysis

Analysis of variance for the relative expression levels of wax biosynthesis-related genes and chemical contents of waxes in different genotypes and genotype × leaf positions were analyzed via one-way and two-way analyses of variance, respectively, using MINITAB 17 statistical software (Minitab, Inc., State College, PA, USA). For pairwise comparisons of means, Tukey’s procedure of separating the means was followed. A principal component analysis was conducted between relative expression of wax biosynthesis genes at leaf position 4 in Experiment 2 and chemical composition of wax compounds measured at the same leaf position. A one-way analysis of variance was conducted for genotypic variation in scores of the first three principal components (PCs).

## Results

### Morphology of Wax Crystals Varies between Low-Wax and High-Wax Cabbage Lines during Leaf Development

**FIGURE 2:**
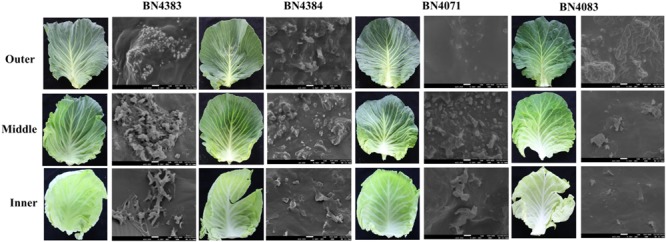
**Cuticular wax phenotype at outer, middle and inner leaves collected from 5-month-old plants of four cabbage inbred lines.** Comparison between epicuticular wax crystals on abaxial cabbage leaf surface of different leaves at 10000x resolution (SEM images, right) from each leaf sample (left) are shown.

At the beginning of experiment, four cabbage lines were categorized into two groups based on visual appearance of leaf wax formation on the leaf surface (Supplementary Figure S1). At the seedling stage, wax formation on the leaf surfaces of high-wax lines was apparent at 4 weeks after sowing in Experiment 2 (data not shown). Two high-wax lines BN4383 and BN4384 had a distinctive whitish appearance on the leaf surface compared to two low-wax lines, BN4071 and BN4161, at 6 weeks of age (Supplementary Figure S1). Images obtained from SEM showed clear differences between high-wax and low-wax lines in wax structures at the abaxial surfaces of the leaves of 5-month-old plants in Experiment 3 (**Figure 2**). Generally, wax crystals in the high-wax lines were dense and prominent compared to those of the low-wax lines. The wax-layers at the leaf surfaces of low-wax lines were like thin wax-platelets (**Figure 2**). During SEM observations, we found that these wax-platelets were dispersed on the leaf surface and localized only in particular locations (see 1000x observations in Supplementary Figure S5). By contrast, wax crystals at the leaf surface of high-wax lines formed densely distributed granular or tubular structures (**Figure 2**; Supplementary Figure S5). Morphology of wax crystals at the abaxial leaf surface changed during leaf development (**Figure 2**; Supplementary Figure S5). Generally, wax deposition was lower in the inner leaves compared to the outer leaves examined (**Figure 2**; Supplementary Figure S5).

### C_29-_hybrocarbon Compounds Predominate in Cabbage Waxes

**FIGURE 3:**
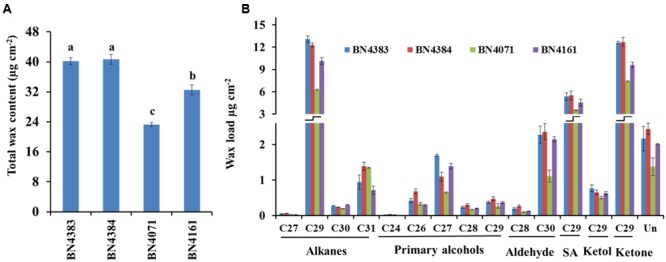
**Cuticular wax content (A)** and composition **(B)** of four inbred lines of *Brassica oleracea*. Cuticular wax was extracted from the leaf position 4 of 10-week-old cabbage plants. Each value is the mean of four independent measurements. Vertical bars indicate standard deviation of means. Different letters (a, b, c) indicate statistically significant variation. SA, secondary alcohol; Un, unidentified compounds.

The total amount of wax in the leaf position 4 of plants at about 20 days of age differed greatly between high-wax and low-wax lines (**Figure 3**; Supplementary Table S3). The total wax per unit area was about twofold higher in the highest-wax line BN4384 compared to the lowest-wax line BN4071 (**Figure 3**). Similarly, the total amount of wax per unit fresh weight was the highest in line BN4384 followed by BN4161 and BN4383 (Supplementary Figure S6). GC–MS analysis identified six different groups of chemicals in the wax crystals collected from all lines (**Figure 3**). These components include alkanes (about 34%), primary alcohols (about 6%), secondary alcohol (about 14%), ketol (about 2%), ketone (about 31%), and aldehydes (about 6%) (**Figure 3**; Supplementary Table S2). The wax composition was dominated by C_29_-alkanes, -ketones, and -secondary alcohols in both high-wax and low-wax cabbage lines (**Figure 3**; Supplementary Figure S6). However, the content of these three C_29_ compounds was significantly higher in high-wax lines compared to low-wax lines (**Figure 3**; Supplementary Table S3). The highest-wax line BN4383 contained strikingly more C_29_ alkane, C_27_ primary alcohol, C_30_ aldehyde, and C_29_ ketone compared to the lowest-wax line BN4071 (**Figure 3**; Supplementary Table S3). In all lines, C_29_ carbons predominated among the alkanes, secondary alcohols, and ketones, whereas C_27_ and C_30_ carbons predominated among the primary alcohols and aldehydes, respectively. The detected alkanes included four compounds with carbon atoms ranging from C_27_ to C_31_, except C_28_ (**Figure 3**). The primary alcohol profile included compounds with the chain length of C_24_ and from C_26_ to C_29_. Aldehyde was composed of C_28_ and C_30_ compounds (**Figure 3**). Secondary alcohol, ketone and ketol contained only C_29_ compounds (**Figure 3**). C_29_ alkane and C_29_ ketone appeared to be particularly important in the formation of wax in leaves (Supplementary Table S2).

### Wax Biosynthetic Genes Become Functional at the First-Formed True Leaf at the Seedling Stage

**FIGURE 4:**
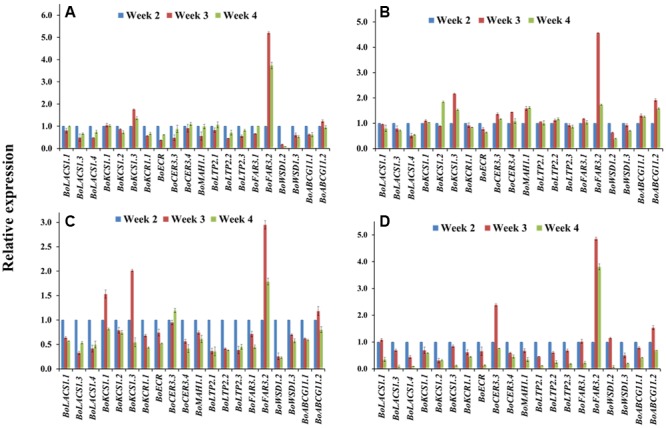
**Relative expression of cuticular wax biosynthetic genes at seedling stage for four cabbage inbred lines.** Expression was analyzed in two high-wax lines, BN4383 **(A)** and BN4384 **(B)**, and two low-wax lines, BN4071 **(C)** and BN4161 **(D)**. Analyzed samples were from the first true leaves collected from plants at 2, 3, and 4 weeks of age. The error bars represent the standard deviation of the means. Values were normalized to those of the 2-week-old plants.

At the seedling stage, qPCR examination was conducted to compare the transcript levels of 20 cuticular wax biosynthetic genes in the first-formed leaves collected (Supplementary Figure S1) from 2-, 3-, and 4-week-old plants of selected cabbage lines (**Figure 4**). The approximate age of the first-formed leaf at the sampling times was 4, 11, and 18 days, respectively. In the high-wax lines, BN4383 and BN4384, a large set of genes such as *BoLACS1.1, BoKCS1.1, BoCER3.4, BoLTP2.1*, and *BoABCG11.2* displayed similar level of expression from week 2 to week 4 in the first-formed true leaves (**Figures 4A,B**), although some of genes, such as *BoLACS1.4, BoKCR1.1, BoECR, BoWSD1.2*, and *BoWSD1.3* were down-regulated in either the lines with increasing leaf age (**Figures 4A,B**; Supplementary Table S4). One notable observation across four genotypes was that the gene *BoFAR3.2* showed a 3- to 5-fold increase in expression from week 2 to week 3 and then decreased expression from week 3 to week 4 (**Figure 4**; Supplementary Table S4). By contrast, majority of genes in the low-wax line BN4071 and BN4161 lowered their expression levels in the week 3 and/or week 4 compared to week 2 (**Figures 4C,D**; Supplementary Table S5), except that a few genes such as *BoKCS1.1, BoKCS1.3*, and *BoFAR3.2* in BN4071, and *BoCER3.3* in BN4161 exhibited higher expression at week 3 compared to week 2 (**Figure 4**; Supplementary Table S5). These data are evident that in all cabbage lines, wax biosynthetic genes became activated in the first-formed leaf at an early age, but that the high-wax line produced higher transcript levels for a comparatively longer period compared to the low-wax lines. At the genotypic level, the relative expression of the 20 genes showed an irregular pattern and none of them exhibited consistently high or low expression in four genotypes across three time points (Supplementary Figure S7). Uniquely, *BoWSD1.2* had strikingly higher expression in BN4161 (Supplementary Figure S7).

### The Expression of Wax Biosynthetic Genes Increases during Leaf Development But Declines in the Older Leaves

**FIGURE 5:**
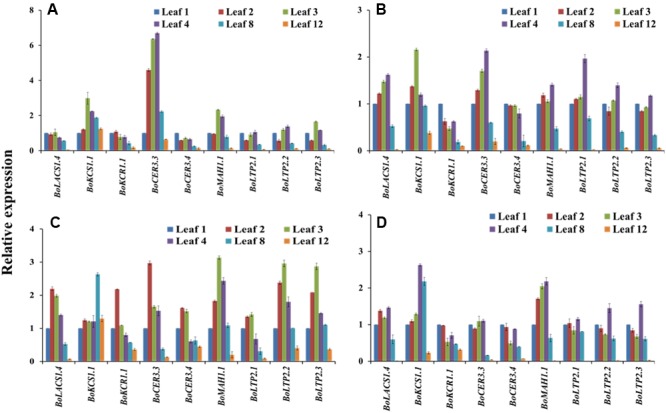
**Relative expression of cuticular wax biosynthetic genes at six different leaf positions of four cabbage inbred lines.** Expression was analyzed in two high-wax lines, BN4383 **(A)** and BN4384 **(B)**, and two low-wax lines, BN4071 **(C)**, and BN4161 **(D)**. The error bars represent the standard deviation of the means. Values were normalized to those of ‘leaf position 1.’

We reasoned that studying the expression of genes in leaves at various developmental stages along the stem axis might provide information to complement measurements of the expression at various time points of a particular leaf throughout its life cycle. The relative expression of nine selected genes related to wax biosynthesis was investigated in leaves at positions 1, 2, 3, 4, 8, and 12 from 10-week-old plants (Supplementary Figure S2). The analyzed genes were from six different super-families and majority of those genes had significant variation for either genotypic or leaf age × genotype interaction in Experiment 1. The plants generally had around 12 live leaves at the time of destructive sampling. The approximate ages of the aforementioned leaves were 5, 10, 15, 20, 40, and 60 days, respectively at the sampling time (Supplementary Figure S2). All nine genes had significantly lower relative expression in the two oldest leaf positions (leaf positions 8 and 12) compared to the younger leaves at positions between 2 and 4, except *BoKCS1.1* in BN4071 and BN4161 (**Figure 5**; Supplementary Table S5). A few genes showed increasing expression in the leaves from position 1 to position 4, with a decrease in older leaves, for example *BoCER3.3* in BN4383 and BN4384; *BoLAS1.4* in BN4384; *BoKCS1.4* and *BoMAH1.1* in BN4161 (**Figure 5**; Supplementary Table S4). Expression of other genes increased from leaf position 1 to leaf position 3 gradually, for example: *BoKCS1.1* in BN4383 and BN4384, *BoMAH1.1, BoLTP2.1, BoLTP2.2*, and *BoLTP2.3* in BN4071 (**Figure 5**). Three other genes, *BoLACS1.1, BoKCR1.1* and *BoCER3.3*, in BN4071 had the highest level of expression in leaf position 2 and their expression declined afterward with leaf age (**Figure 5**). When the four genotypes were compared, expression for majority of the genes (except *BoLTP2* genes) was significantly higher in high-wax lines than in the low-wax lines (Supplementary Figure S8). Three *BoLTP2* genes had higher expression in low-wax line BN4071 (Supplementary Figure S8). In general, transcript levels of wax biosynthetic genes showed an increasing trend up to 15–20 days of age and declined greatly in the older leaves during leaf development.

### Expression of Wax Biosynthetic Genes Differed Greatly between the Inner and Outer Leaves in the Heads of 5-month-old Cabbage Plants

**FIGURE 6:**
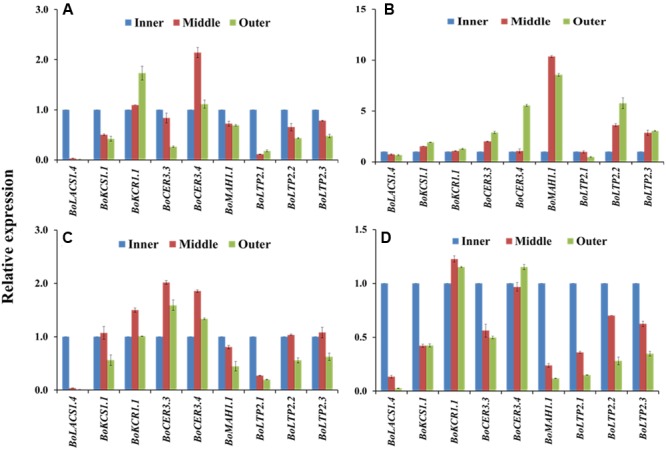
**Relative expression of cuticular wax biosynthetic genes at the outer, middle, and inner leaves of four cabbage inbred lines.** Expression was analyzed in two high-wax lines, BN4383 **(A)** and BN4384 **(B)**, and two low-wax lines, BN4071 **(C)** and BN4083 **(D)**. The error bars represent the standard deviation of the means. Values were normalized to those of the ‘inner leaf position.’

The four cabbage lines displayed notable variation in expression of wax biosynthesis-related genes in the outer leaves compared to the inner leaves of cabbage heads (**Figure 6**). The majority of the genes including *BoKCS1.1, BoKCR1.1, BoCER3.3, BoCER3.4, BoLTP2.2, BoLTP2.2*, and *BoMAH1.1* were upregulated in the outer leaves compared to the inner leaf in BN4384 (**Figure 6**; Supplementary Table S4), except that *BoLACS1.4* and *BoLTP2.1* were down-regulated from the inner to the outer leaves (**Figure 6**). By contrast, in another high-wax line, BN4383, seven genes (*BoLACS1.4, BoKCS1.1, BoCER3.3, BoMAH1.1, BoLTP2.1, BoLTP2.2*, and *BoLTP2.3*) had lower expression in the outer leaves compared to the inner leaf, and only two genes (*BoKCR1.1* and *BoCER3.4*) showed higher expression in the outer leaves (**Figure 6A**). Similar to the line BN4383, in two low-wax lines the majority of the genes had lower expression in the outer leaves compared to the inner leaf (**Figure 6**), except that *BoKCR1.1, BoCER3.3*, and *BoCER3.4* in BN4071 had the higher level of expression in the middle and/or outer leaves (**Figure 6**). At the genotype level, aside from *BoKCR1.1*, the other eight genes had remarkably lower expression in BN4384 at the inner leaf position compared to the other three lines (Supplementary Figure S9). Moreover, *BoLTP2.2* and *BoLTP2.3* genes had notably higher expression in BN4083 in all three leaf positions, i.e., in outer, middle, and inner leaves (Supplementary Figure S9; Supplementary Table S5). These data indicate that, except for the highest wax line, in general, the levels of transcripts of most wax biosynthetic genes are decreased from the inner to the two outer leaves.

### BoCER was Expressed Highly in High-Wax Lines But BoLTP was Expressed Highly in the Lowest-Wax Line

Principal component analysis revealed a significant association between relative expression of wax biosynthetic genes and levels of chemical compounds within wax crystals in leaf position 4 of 10-week-old cabbage inbred lines (**Table 3**). PC1 explained 47.9% of the data variation (**Table 3**). The coefficients of PC1 indicated a positive association between the expression levels of three *BoLTP2* genes and the contents of C_31_ alkane, C_24_ and C_26_ primary alcohols, C_29_ secondary alcohol, and C_29_ alkane (**Table 3**). PC1 also indicated a positive association between expression of *BoCER3.4* and contents of C_29_ alkane, C_27_ primary alcohol, and C_29_ ketol (**Table 3**). The mean PC scores indicated a contrast between BN4071 and BN4161 for the two largest contrasting PC scores (**Table 3**). These two lines had significant differences in the contents of total wax, C_29_ alkane, C_27_ primary alcohol and C_29_ ketol (**Figure 3**). In addition, expression of *BoCER3.4* in BN4161 was strikingly higher at leaf position 4 compared to BN4071 (Supplementary Figure S8). PC1 further revealed a contrasting association between the expression levels of *BoCER3.4* and *BoLTP2* in the lowest-wax line compared to the other three lines (**Table 3**; Supplementary Figure S8). PC2 explained 22.8% of the data variation (**Table 3**). PC2 highlighted the significant contrast between the two high-wax lines and the low-wax lines in terms of expression of some notable genes and the levels of wax compounds (**Table 3**). PC2 had comparatively larger and positive coefficients for *BoLACS1.4, BoLTP2.2*, C_30_ alkane and C_29_ secondary alcohol but comparatively lower and negative coefficients for *BoCER3.3*, C_28_ aldehyde and C_26_ primary alcohol for contrasting mean PC scores between low-wax and high-wax lines (**Table 3**). PC3 accounted for 13.8% of the data variation largely due to variation in expression levels of *BoKCS1.1* and contents of C_29_ primary alcohol, C_30_ aldehyde and C_29_ ketone in between cabbage lines BN4384 and BN4161 (**Table 3**). Overall, the higher expression of *BoCER* and a few other genes increased the wax content, but the higher expression of *BoLTP* genes in the lowest-wax line apparently did not.

**Table 3:** Principal component analysis of genotypic variation in wax component levels and relative expression of wax biosynthesis-related genes in four cabbage inbred lines of *B. oleracea.*

Variable	PC1	PC2	PC3
*BoLACS1.4*	-0.123	0.314	0.254
*BoKCS1.1*	-0.103	-0.101	-0.465
*BoKCR1.1*	0.154	-0.021	-0.228
*BoCER3.3*	0.084	-0.395	-0.090
*BoCER3.4*	-0.275	0.086	-0.109
*BoMAH1.1*	0.143	0.195	-0.221
*BoLTP2.1*	0.250	0.161	-0.130
*BoLTP2.2*	0.222	0.216	0.106
*BoLTP2.3*	0.252	0.132	0.191
C_27_ alkane	0.246	-0.194	0.199
C_29_ alkane	-0.269	-0.180	0.049
C_30_ alkane	-0.046	0.376	-0.202
C_31_ alkane	0.279	0.126	0.065
C_24_ PA	0.267	-0.130	-0.066
C_26_ PA	0.245	-0.235	-0.048
C_27_ PA	-0.276	0.139	0.038
C_28_ PA	0.246	-0.080	-0.195
C_29_ PA	0.131	0.024	-0.383
C_28_ Aldehyde	0.078	-0.396	-0.001
C_30_ Aldehyde	-0.203	-0.100	-0.290
C_29_ SA	0.172	0.254	-0.109
C_29_ Ketol	0.240	0.195	-0.195
C_29_ Ketone	0.202	-0.063	0.347
% variation explained	47.9	22.8	13.8
p (genotype)	<0.001	<0.001	<0.001

**Genotype**	**Mean PC scores (±SE)**		

BN4383	1.685 ± 0.40	-3.291 ± 0.36	-0.931 ± 0.34
BN4384	-2.737 ± 0.40	-4.24 ± 0.36	2.358 ± 0.34
BN4071	4.302 ± 0.40	2.145 ± 0.36	0.515 ± 0.34
BN4261	-3.350 ± 0.40	1.570 ± 0.36	-1.942 ± 0.34

*PC, principal component; p, statistical significance; PA, primary alcohol; SA, secondary alcohol.*

## Discussion

The present study was undertaken to understand the morphology, biochemistry, and genetics of wax formation in cabbage. In this study, we investigated the relative expression of wax biosynthetic genes at different developmental stages of leaves in three different growth stages of cabbage plants. We also identified and quantified the biochemical compounds present in the cabbage leaf and investigated their relationship with the expression levels of wax biosynthesis-related genes at the same developmental stages. Furthermore, we explored the pattern and variability of wax crystals produced in low- and high-wax cabbage lines. Data obtained from three different experiments (**Table 1**) provide a clear understanding of the relationships between wax biosynthesis-related gene expression and wax deposition in cabbage lines.

### Wax Crystal Types and Sizes Vary during Wax Leaf Development in Different Genotypes

We investigated the morphology of wax crystals on the abaxial sides of leaf blades by SEM in cabbage leaves collected from 5-month-old plants. Wax deposition started from the inner leaf and the size and density of cuticular wax crystals increased from the inner to the middle leaves in all cabbage lines (**Figure 2**). These observations highlight that wax deposition on the leaf surface changes with leaf development in cabbage plants. The density and size of wax crystals were greater in high-wax lines than in low-wax lines (**Figure 2**). Consistent with our results in cabbage, bloomed (waxy) leaves of sorghum and wheat have dense cuticular wax crystals, whereas bloomless (non-waxy) leaves have fewer wax crystals (Tarumoto, 2005; Wang et al., 2015).

### Wax Content is Related to Wax Crystals

In *Arabidopsis*, there is 10-fold more stem wax than leaf wax, and wax crystals are visible on the stem but not on leaves (Lee et al., 2009a). In this study, wax crystals were observed in inner, middle and outer leaves of 5-month-old plants (**Figure 2**; Supplementary Figure S5) and wax composition and content was measured in 20-days-old leaves from 10-week-old plants (**Figure 3**). The presence of markedly larger wax crystals in the high-wax lines might be associated with the higher wax content of these plants (**Figure 2**). Moreover, the higher total wax content per unit leaf area is attributable to larger and denser wax-crystals per unit leaf area in the high-wax lines compared to low-wax lines (**Figures 2** and **3**). By contrast, the lower total wax content observed in a high-wax line, BN4383, on a per unit fresh weight basis compared to a low-wax line, BN4161, can probably be explained by variation in leaf thickness, water content, and other morphological attributes including size and density of wax crystals between these two cabbage lines (**Figure 3**; Supplementary Figure S6).

### C_29_ Compounds Are Predominant in Cabbage Wax Crystals

The dominance of C_29_ alkane in cabbage leaf wax is very similar to that in broccoli (**Figure 3**; Lee et al., 2015), *Nicotiana* (Chiang and Grunwald, 1976), *Arabidopsis* (Jenks et al., 1995; Bernard and Joubès, 2013), and *Rosa* (Jenks et al., 2001). By contrast, alkanes are very low or undetectable in wax of barley and maize (Post-Beittenmiller, 1996). The high proportion of C_29_ ketone in cabbage leaf wax is also similar to that of broccoli (Lee et al., 2015), and the stem and siliques, but not leaves, of *Arabidopsis* (Lee et al., 2009a). We found six chemical compounds in the cabbage waxes: alkanes, primary alcohols, aldehydes, secondary alcohols, ketols and ketones, but fatty acid was not present (Supplementary Table S2). In contrast to cabbage, low-wax broccoli contained 13.5% and high-wax broccoli contained 15.3% fatty acids (Lee et al., 2015), leaves and stems of wild-type *Arabidopsis* contained 8.36 and 1.37% (Lu et al., 2012), and leaves of wheat contained 0.5–11.0% fatty acids (Wang et al., 2015). In the present study, we observed dominant alkane chain lengths of C_27_, C_29_, and C_31_, similar to leaves of broccoli (Lee et al., 2015) and stems, leaves and siliques of *Arabidopsis* (Lee et al., 2009a); by contrast, C_30_ alkane appears to be a unique component in cabbage compared to broccoli (**Figure 3**; Lee et al., 2015). The presence of C_29_ secondary alcohol in cabbage leaf wax is similar to that of broccoli, and stems and siliques of *Arabidopsis* (Lee et al., 2009a, 2015). Among the primary alcohols (**Figure 3**), C_24_, C_26_, and C_28_ primary alcohols are also present in broccoli and *Arabidopsis* (Lee et al., 2009a, 2015) but C_27_ and C_29_ primary alcohols are uniquely found in cabbage (**Figure 3**; Lee et al., 2009a, 2015). Similar to our results for cabbage, C_28_ and C_30_ aldehydes are present in stems, leaves and siliques of *Arabidopsis* and in broccoli (Lee et al., 2015). Broccoli, however, also contains C_24_ and C_26_ aldehydes (Lee et al., 2015), which were absent in cabbage (**Figure 3**; Lee et al., 2009a).

### Wax Biosynthetic Genes Are Differentially Expressed during Leaf Development in High- and Low-Wax Cabbage Lines

In both high- and low-wax lines, wax biosynthetic genes were expressed in the first-formed leaves of around 4 days of age (collected from 2-week-old plants), indicating that wax biosynthesis starts at the appearance of new leaves (**Figure 4**). A decrease in gene expression in the low-wax lines at 11- and 18-day-old leaves (collected from 3- and 4-week-old plants) indicated that wax formation in those lines declined with leaf aging (**Figure 4**). However, in the high-wax line BN4384, a consistent level of gene expression with leaf age up to 18 days indicated that wax formation did not decline as leaves aged, at the early stage (**Figure 4**). When gene expression was compared between genotypes, most of the genes had lower expression in the first-formed leaf collected from 3- and 4-week-old plants in the low-wax line, which further indicated that wax deposition declined at the early leaf age in those lines (**Figure 4**; Supplementary Figure S7).

**FIGURE 7:**
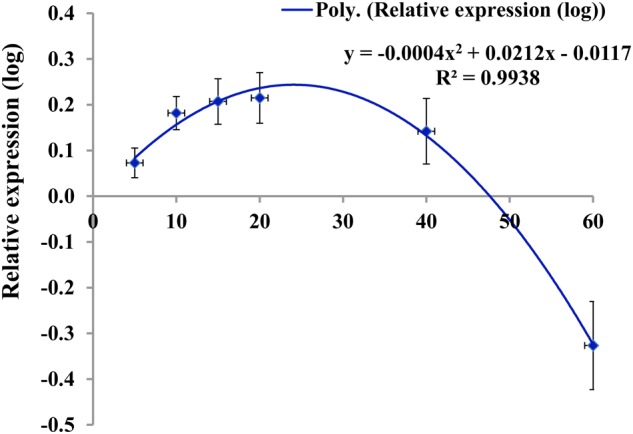
**Pattern of expression of wax biosynthetic genes (log-transformed relative expression values of un-weighted average across nine genes) in four cabbage lines in relation to leaf age at different leaf positions.** Data fit a quadratic polynomial. Leaf 1 was the youngest leaf. Tentative ages of leaves 1, 2, 3, 4, 8, and 12 were approximately 5, 10, 15, 20, 40, and 60 days. Vertical bars indicate standard error of the mean for each leaf position.

When the relative expression of nine wax biosynthetic genes was averaged across six different leaf positions studied from 10-week-old plants, the average expression level of all nine genes fit well to a quadratic curve (**Figure 7**). The average expression in the two older leaf positions (leaf positions 8 and 12) was strikingly lower compared to the four younger leaves (**Figure 5**; Figure S8), suggesting that wax biosynthesis greatly declines in the older leaves before senescence. This assumption seems valid for both the low- and high-wax lines. At the genotype level, the strikingly higher expression of *BoCER3.3* in the two high-wax lines compared to the two low-wax lines suggested that this particular gene might play a significant role in formation of the larger and denser wax crystals found in two high-wax lines (Supplementary Figure S8). However, this speculation is a subject for further investigation, particularly to address how *BoCER3.3* brings about biochemical changes in high-wax lines to accumulate larger and denser wax crystals compared to low-wax lines. In *Arabidopsis, Cer3* mutants accumulate dramatically reduced levels of aldehydes, alkanes, secondary alcohols, and ketones compared to wild-type plants (Jenks et al., 1995; Rowland et al., 2007). It was speculated that in *Arabidopsis*, the *CER3* gene product might release fatty acids from elongase complexes (Jenks et al., 1995). In line with previous results, our study recorded a decrease of 52% C_29_ alkane, 41% C_29_ ketone, 34% C_29_ secondary alcohol and 51% C_30_ aldehyde in the lowest-wax line, BN4071, compared to the highest-wax line, BN4384 (**Figure 3**). In 5-month-old plants, a decreasing trend in expression of wax biosynthetic genes with leaf aging except the in highest-wax line BN4384 (**Figure 6**) indicated that wax formation declined with leaf aging except in that particular line. A generally increasing trend in expression of wax biosynthetic genes in BN4384 from the inner leaf to outer leaves suggests that wax formation remained active with leaf aging in this line, in which wax is heavily deposited at the outermost leaves (**Figure 6**).

### BoCER Expression Significantly Increases Wax Deposition in High-Wax Lines

The mean of the PC1 scores indicated that the larger proportion of the variation explained by PC1 was due to higher expression *BoLTP2* genes in the BN4071 line compared to the other three lines (**Table 3**; Supplementary Figure S8). That same line had the lowest level of C_29_ secondary alcohol and ketone (**Figure 3**). Based on these results for PC1, it seems likely that the higher level of expression of *BoLTP2* genes, which are involved in transfer of ketones to lipid transfer proteins, is related to the reduced wax deposition in that lowest-wax line (BN4071) (**Figure 1**; Supplementary Figure S8). The larger coefficients of PC1 further suggested that *BoCER3.4* is actively involved in wax formation, likely via its role in converting aldehydes to alkanes (**Figure 1**). The association captured by the larger coefficients of PC2 indicated that the higher expression of *BoCER3.3* in high-wax lines likely enhanced the biosynthesis of C_28_ aldehyde in the high-wax lines compared to low-wax lines (**Figure 3**; Supplementary Figure S8). A relatively higher accumulation of C_29_-alkane, C_29_-secondary alcohol and C_29_-ketone in high-wax lines with corresponding higher expression of *BoCER3* genes indicated that these genes probably catalyzed VLCHCs, including aldehydes, to alkanes at a higher rate in high-wax lines compared to low-wax lines (**Figures 1** and **3**). Rowland et al. (2007) observed major reductions of aldehydes, alkanes, secondary alcohols, and ketones in *Arabidopsis cer3* mutants along with a significant increase in C_30_ primary alcohol, indicating that *CER3* is a key gene for the decarbonylation pathway in wax biosynthesis.

In Experiment 1, the *BoKCS1, BoKCR1, BoFAR3*, and *BoWSD1* genes were markedly downregulated in the low-wax lines with increasing leaf age compared to high-wax lines in the first-formed leaves (**Figure 4**). When the plants from same batch attained 6 weeks of age, apparent wax content on leaf surface was much lower in the low-wax lines compared to high-wax lines, indicating that decreases in expression of those wax biosynthetic genes reduced wax accumulation in low-wax lines (Supplementary Figure S1). The declined levels of expression of *BoKCS1* and *BoKCR1* were probably associated with reduced biosynthesis of acyl-CoAs, which eventually affected total wax biosynthesis and accumulation in low-wax lines. Similarly, expression of three *BoLACS1* genes in comparatively young leaves in Experiment 1 and Experiment 2 was significantly lower in low-wax lines compared to high-wax lines, which might also affect acyl-CoA biosynthesis in low-wax lines and thus affect wax accumulation (Supplementary Figures S7 and S8). In broccoli, expression of *BoLACS1, BoKCS1*, and *BoKCR1* genes was also notably higher in 3- and 10-week-old bloomed plants compared to bloomless plants (Lee et al., 2015). In addition, the *BoMAH1* gene, which is involved in biosynthesis of secondary alcohols and ketones, was expressed highly with increasing leaf age in the highest wax line BN4384 in both Experiment 1 and Experiment 3 (6 to 10-fold) indicating that the decarbonylation pathway remains more active in the high-wax lines for a prolonged period compared to low-wax lines (**Figures 4** and **6**).

In conclusion, the composition of *Brassica* waxes is known to vary depending on environmental stress conditions and on genetic factors. Therefore, in any future investigations, effects of developmental programming must be distinguished from effects of growth conditions. Another point necessary to bear in mind is that leaf positions may affect local light conditions within a cabbage rosette and therefore influence wax composition more than leaf age. The three experiments described here were conducted on plants grown under two different sets of conditions, and results from them can therefore not be interpreted as a function of development alone, i.e., the overall leaf developmental effect on wax accumulation might be also partly affected by experimental conditions.

## Conclusion

The present study was conducted to understand the genetics and biochemistry of wax formation in relation to wax morphology in cabbage inbred lines under optimal growth environments. In this study, expression levels of wax biosynthetic genes were studied at three different developmental stages in cabbage plants. Expression generally increased initially with leaf development and then decreased with leaf age, especially in low-wax lines following a quadratic function. In the highest-wax line, the relative expression of wax biosynthesis genes was consistently higher for a comparatively longer period, which indicated wax deposition at a higher rate for a longer duration. Relative expression of wax biosynthetic genes declined greatly in the older leaves. Wax composition of the cabbage plants was predominated by C_29_ alkanes, secondary alcohols and ketones in both high- and low-wax lines. Principal component analysis revealed that *CER3* has a vital role in the greater wax deposition observed in the highest-wax lines. The high-wax plants generally formed larger and denser wax crystals compared to the low-wax cabbage lines. The results of this study are useful in understanding the genetic underpinnings of wax formation patterns in cabbage.

## Author Contributions

I-SN, J-IP, AR, KY, and RL conceived and designed the study. RL managed the experimental plants, collected samples, prepared cDNA, and conducted qPCR analysis. RL, KY, and AR prepared the samples for SEM analysis. MS and JK analyzed the chemical composition of the wax compounds. RL and AR analyzed the data and wrote the manuscript. RL and KY edited tables and figures. All authors read the final version of the manuscript.

## Conflict of Interest Statement

The authors declare that the research was conducted in the absence of any commercial or financial relationships that could be construed as a potential conflict of interest.

## Footnotes

1 http://www.ncbi.nlm.nih.gov

2 http://www.ocri-genomics.org/bolbase/blast/blast.html

3 http://linux1.softberry.com/berry.phtml

4 http://frodo.wi.mit.edu/primer3
