# Supplementary material for: Developmental and Genotypic Variation in Leaf Wax Content and Composition, and in Expression of Wax Biosynthetic Genes in Brassica oleracea var. capitata
Source: Front Plant Sci. 2017 Jan 9;7:1972. doi: 10.3389/fpls.2016.01972 (PMC5220014; doi:10.3389/fpls.2016.01972)
Supplement: Supplementary file 2 [file Data_Sheet_1.docx]

**Supplementary data**

**
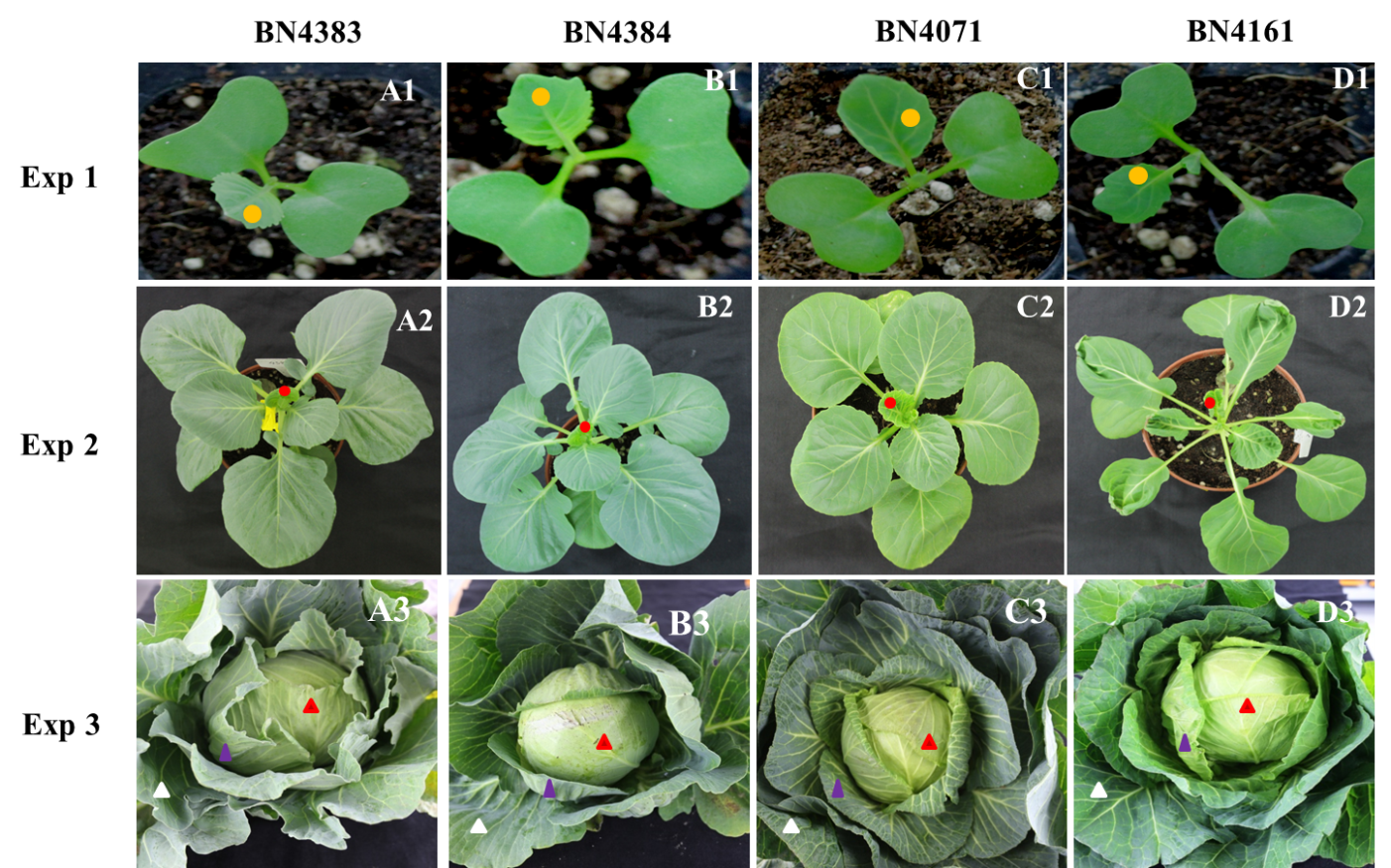
**

**Figure S1.** External appearance of cuticular waxes on leaves of plants of four cabbage lines at two weeks (A1–D1, Experiment 1), six weeks (A2–D2, Experiment 2) and five months (A3–D3, Experiment 3) of age. Orange circles in Experiment (Exp) 1 indicate first-formed true leaves; red circle in Exp 2 indicates the youngest leaf (reference point); the red, violet and white triangles in Exp 3 indicate inner, middle and outer leaf positions.


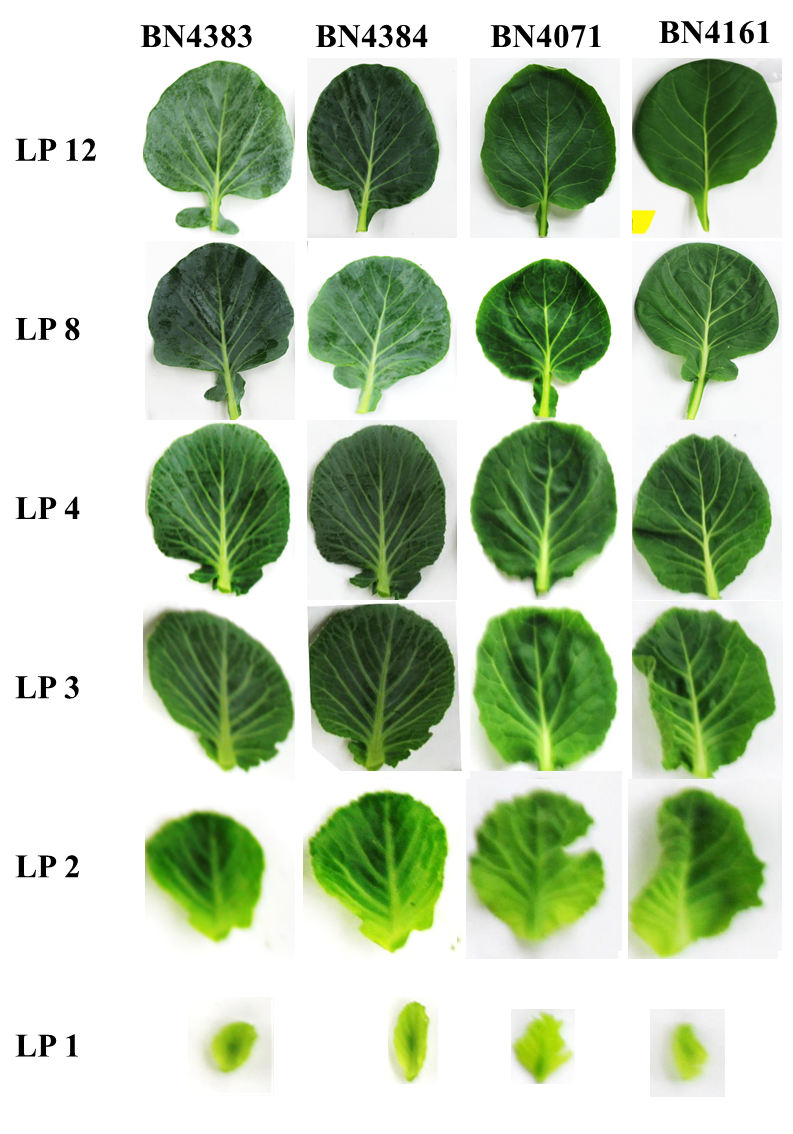


**Figure S2.** External appearance of cuticular waxes on leaves at different leaf positions (LP) from 10-week-old plants of four cabbage lines (Approximate age of leaf at LP 1 was less than 5 days, and that of LP 2, 3, 4, 8 and 12 leaves were 10, 15, 20, 40 and 60 days, respectively).


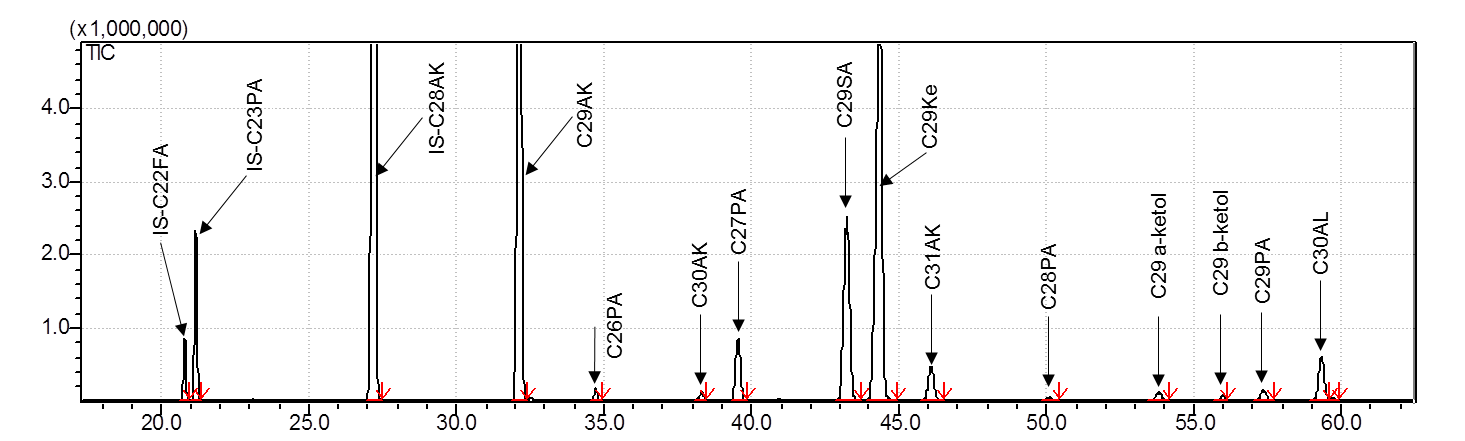


(GC-MS peaks; IS, internal standard)
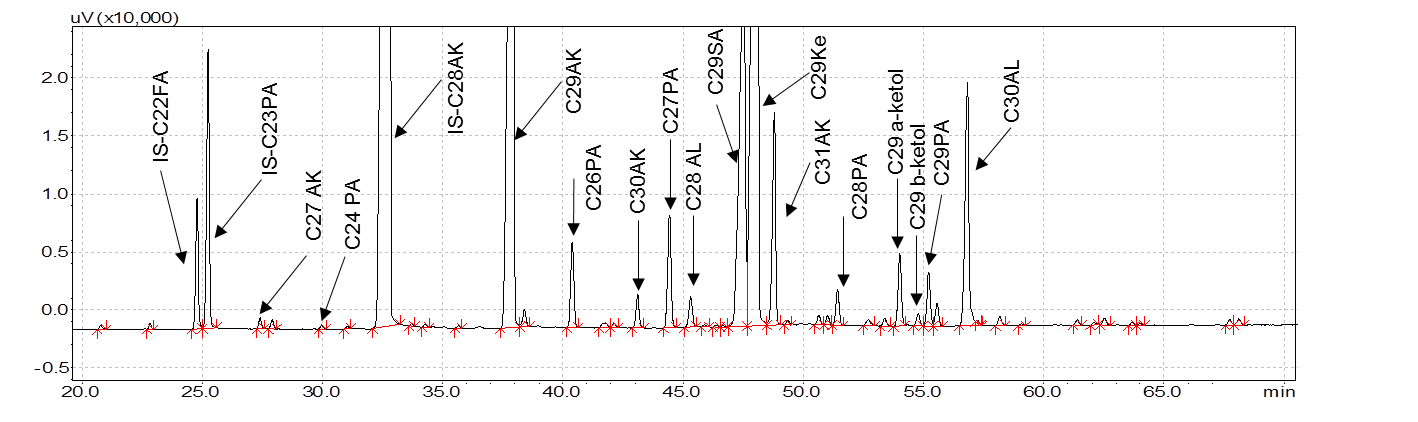


(GC peaks; IS, internal standard)

**Figure S3.** Peaks related to measuring wax compounds in GC-MS and GC. FA, fatty acids; PA, primary alcohol; AK, alkane; SA, secondary alcohol; Ke, Ketone and AL, aldehyde.


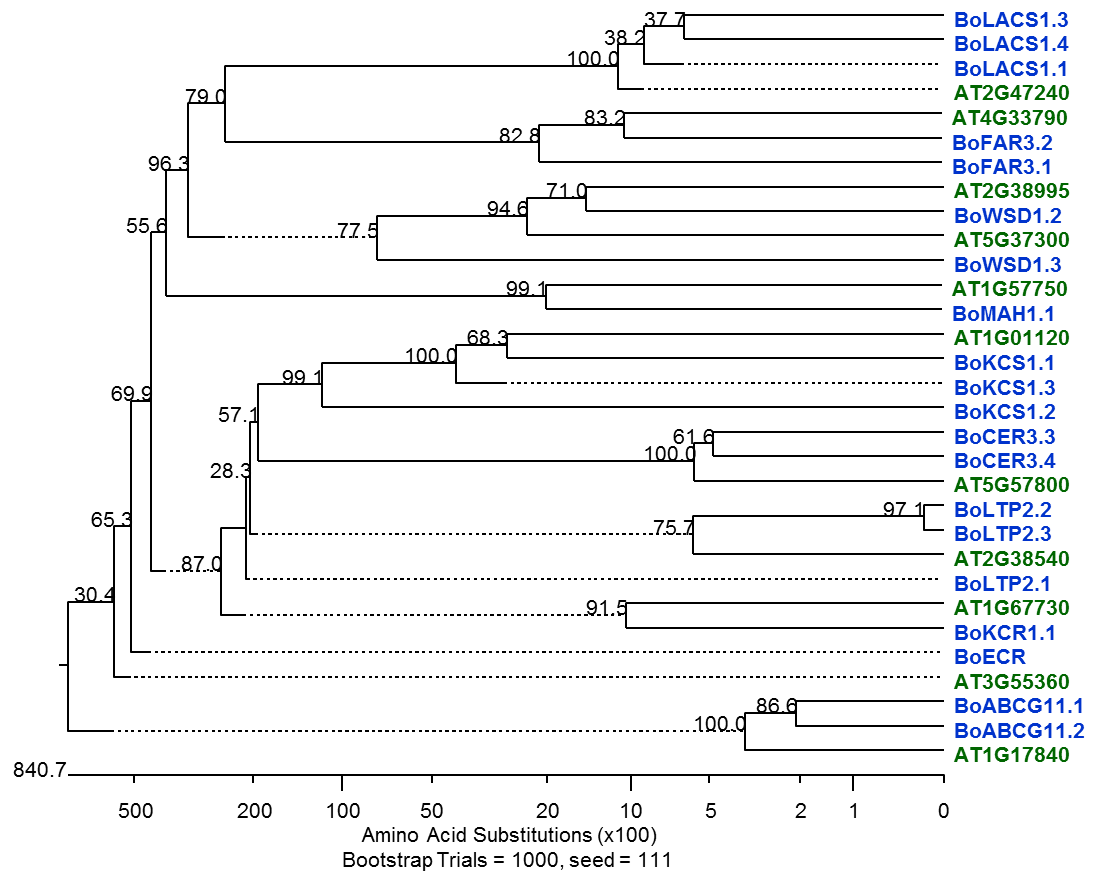


**Figure S4.** Phylogenetic tree indicates clustering between published *Arabidopsis thaliana* wax biosynthetic proteins (green letters) and selected *Brassica oleracea* proteins (blue letters).


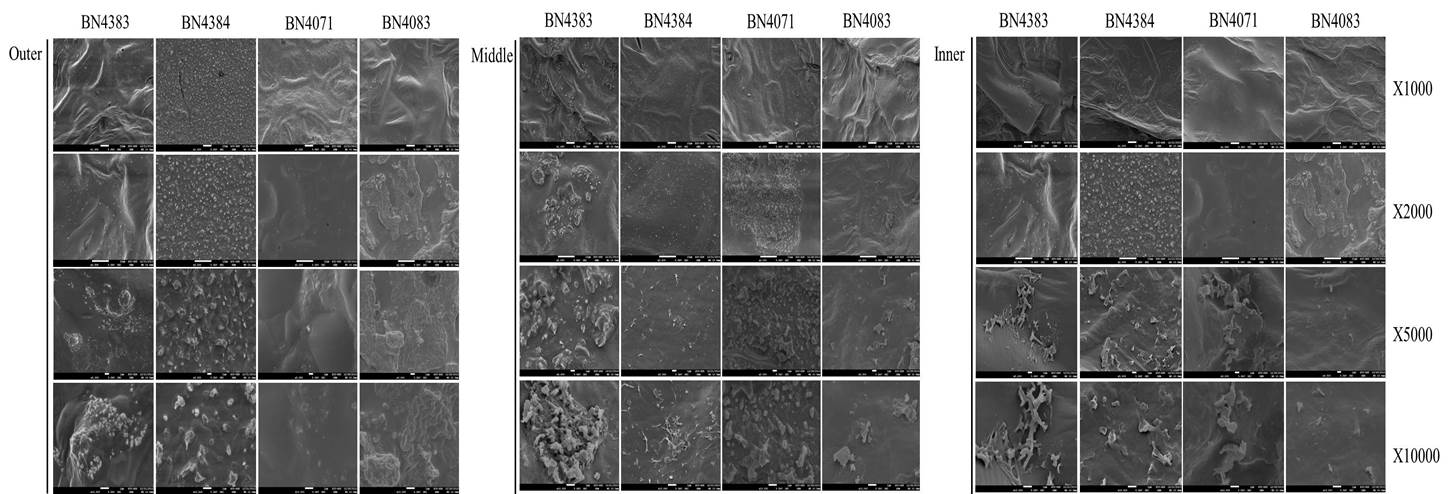


**Figure S5.** Developmental changes in epicuticular wax crystals on the cabbage leaf surface. SEM images are shown at 1000x, 2000x, 5000x and 10000x resolution for four cabbage inbred lines (BN4383, BN4384, BN4071 and BN4083) at inner, middle and outer leaf positions as described in Figure S1.

**
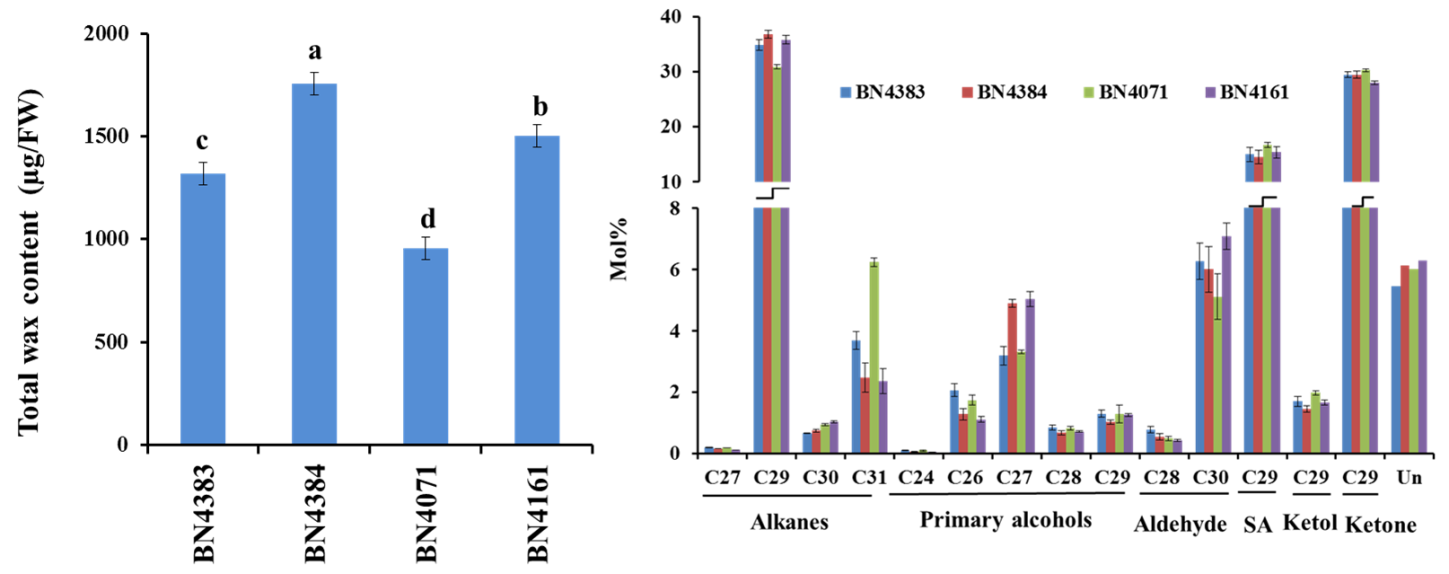
**

**Figure S6**. Cuticular wax content (A) and wax composition (B) in four cabbage inbred lines of *B. oleracea*. Cuticular wax was extracted from leaves of 10-week-old cabbage plants. Each value is the mean of four independent measurements. Vertical bars indicate standard deviation of means. Different letters (a, b, c and d) indicate statistically significant variation. SA, secondary alcohol; Un, unidentified compounds.

**
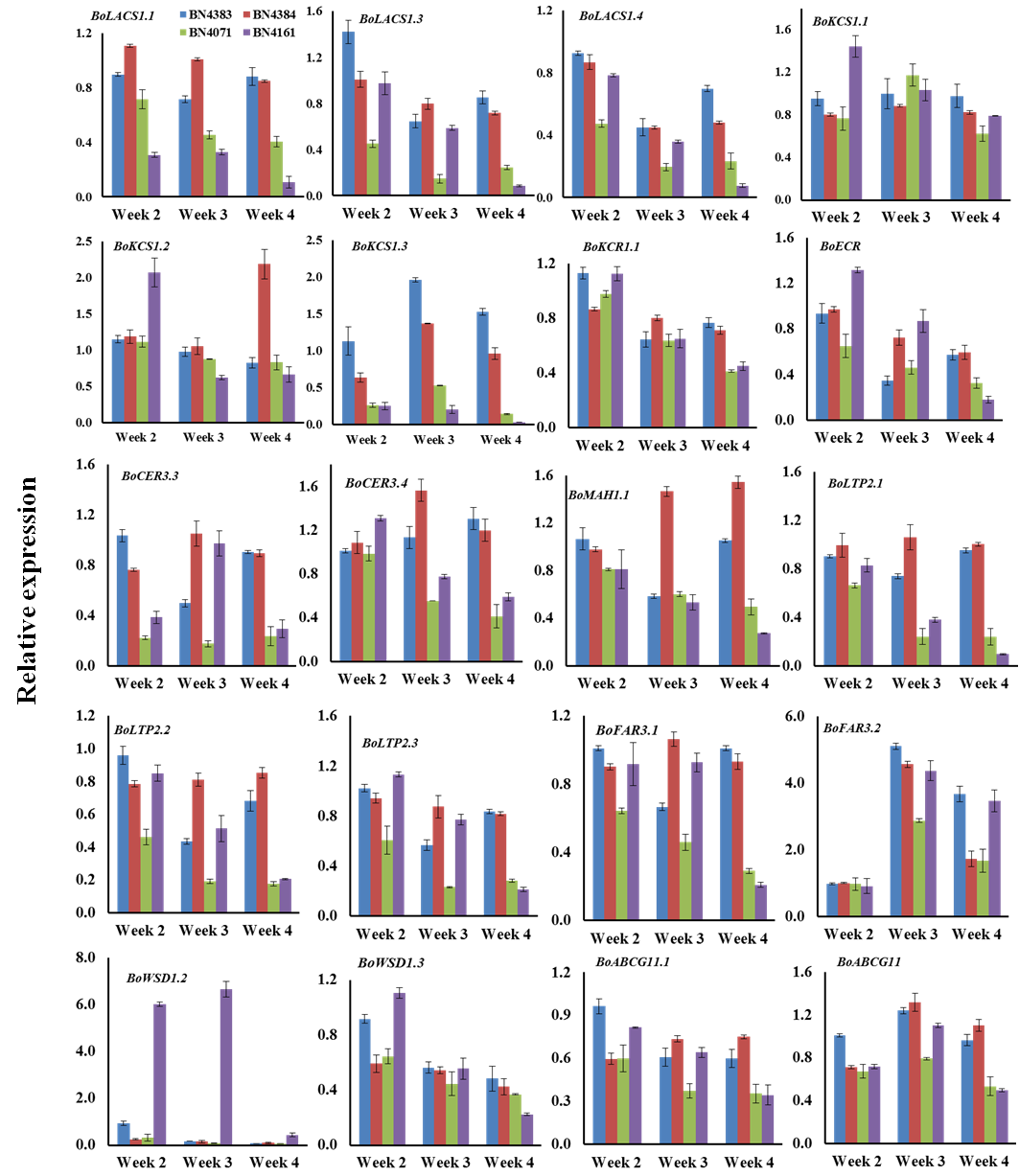
**

**Figure S7.** Genotypic variation in relative expression of wax biosynthesis-related genes in the first true leaf of four cabbage inbred lines collected from two-, three- and four-week-old plants. Approximate leaf age at the three sampling points was 4, 11 and 18 days, respectively, as the first true leaf appeared when the plants were around 10 days of age. Vertical bars indicate standard deviation of means.


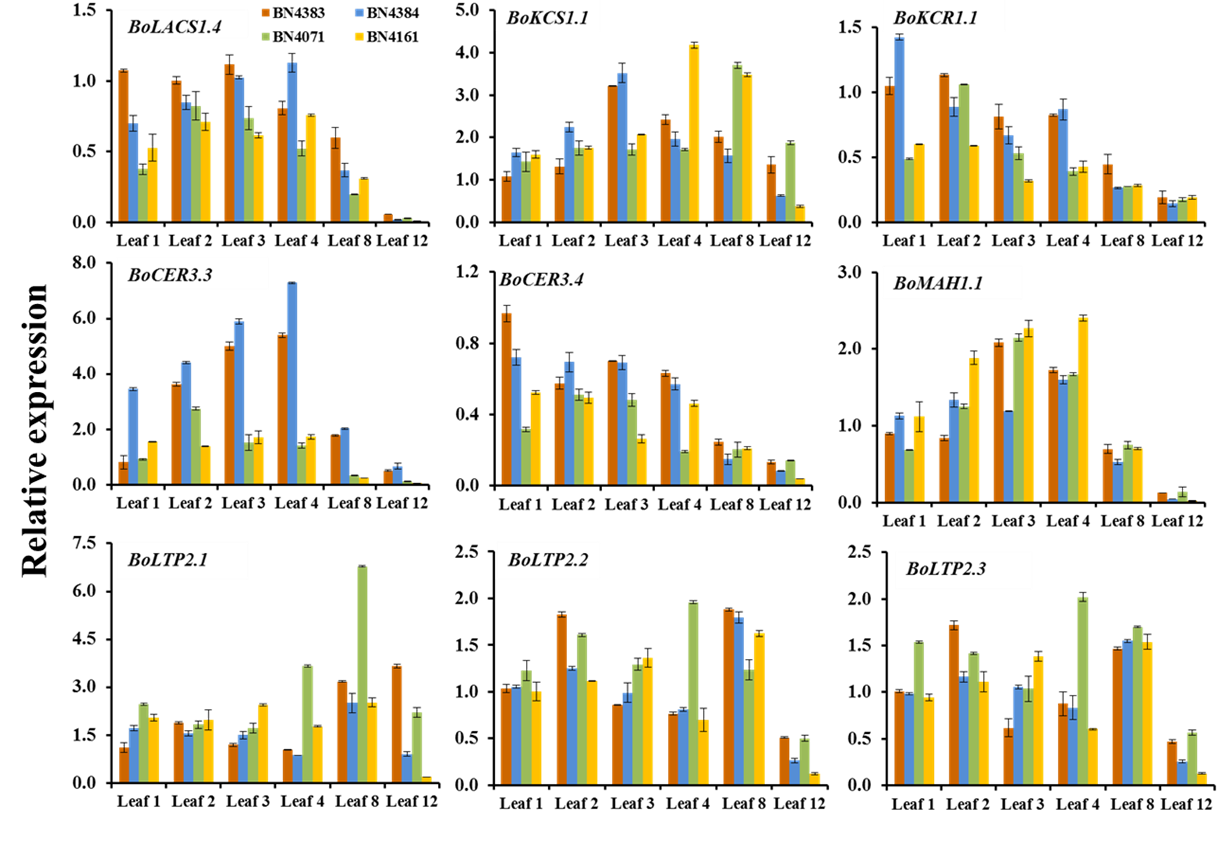


**Figure S8.** Genotypic variation in relative expression of wax biosynthesis-related genes at six different leaf positions of 10-week-old plants in four cabbage inbred lines. Leaf samples were collected by destructive harvesting as shown in Figure S2. Vertical bars indicate standard deviation of means.

**
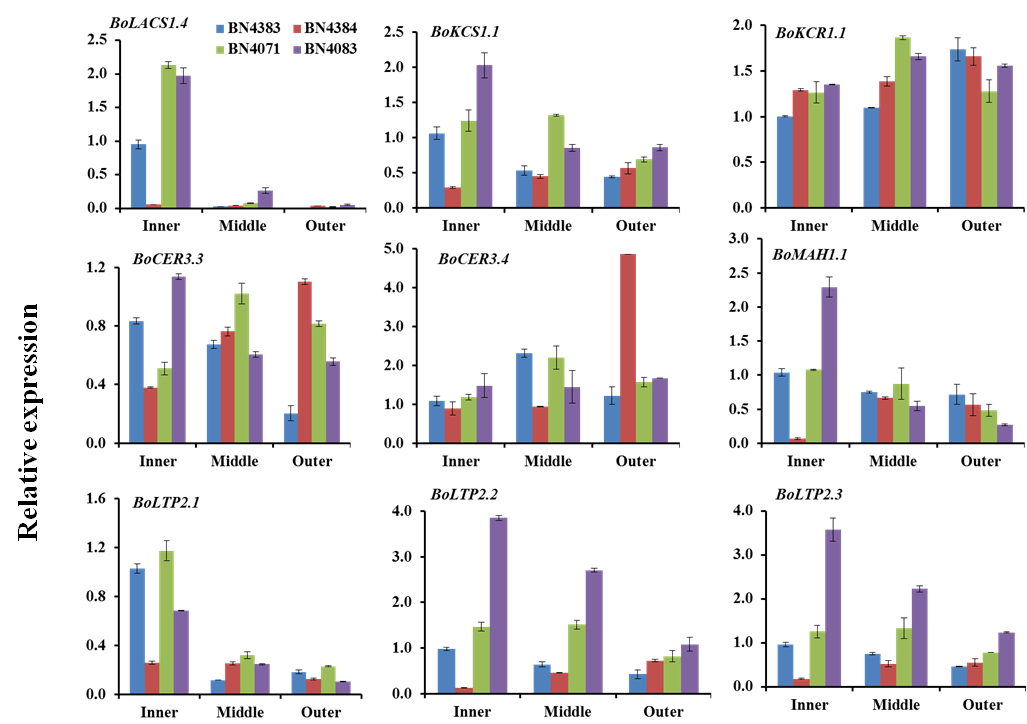
**

**Figure S9.** Genotypic variation in relative expression of wax biosynthesis-related genes at the inner, middle and outer leaf positions of five-month-old plants in four cabbage inbred lines. Vertical bars indicate standard deviation of means.

**Table S1.** Size of the leaves collected from different leaf positions in Experiments 1-3. Data are the average (±sd) of three individual observations.

a) Length and diameter of the first-formed true leaf collected from a two to four weeks old cabbage plants grown in a standard growth chamber conditions in Experiment 1

| Sampling point  (Age of first-formed leaf) | **BN4383** | | **BN4384** | | **BN4071** | | **BN4161** | |
| --- | --- | --- | --- | --- | --- | --- | --- | --- |
|  | Length (cm) | Diameter (cm) | Length (cm) | Diameter (cm) | Length (cm) | Diameter (cm) | Length (cm) | Diameter (cm) |
| Week 2 (4 d) | 4.8±0.4 | 4.0±0.2 | 5.0±0.5 | 4.9±0.4 | 5.2±0.5 | 4.5±0.2 | 3.9±0.3 | 3.7±0.4 |
| Week 2 (11 d) | 9.0±0.6 | 7.5±0.4 | 6.8±0.3 | 7.8±0.6 | 7.9±0.8 | 8.8±0.6 | 6.2±0.9 | 5.9±0.4 |
| Week 2 (18 d) | 12±0.7 | 10±0.8 | 10±0.7 | 8.2±0.6 | 9.7±0.9 | 9.6±0.5 | 9.1±0.3 | 8.0±0.4 |

b) Length and diameter of the leaves at different leaf positions collected from a 10-week-old cabbage plants grown in a standard growth chamber conditions in Experiment 2

| Leaf position  (Leaf age) | **BN4383** | | **BN4384** | | **BN4071** | | **BN4161** | |
| --- | --- | --- | --- | --- | --- | --- | --- | --- |
|  | Length (cm) | Diameter (cm) | Length (cm) | Diameter (cm) | Length (cm) | Length (cm) | Diameter (cm) | Length (cm) |
| 1 (5 d) | 4.3±0.3 | 4.0±0.4 | 4.3±0.4 | 4.3±0.4 | 4.4±0.5 | 4.5±0.1 | 3.5±0.5 | 3.2±0.6 |
| 2 (10 d) | 9.0±0.5 | 6.5±0.6 | 7.0±0.3 | 7.3±0.4 | 7.6±1.5 | 8.2±0.5 | 5.2±1.0 | 5.2±0.3 |
| 3 (15 d) | 11.3±1.5 | 9.1±1.0 | 9.0±0.7 | 7.5±0.6 | 8.7±1.9 | 8.4±0.9 | 8.3±0.3 | 7.4±0.4 |
| 4 (20 d) | 12.5±2.0 | 8.5±0.9 | 11.5±0.4 | 9.8±0.6 | 10.1±1.3 | 9.9±1.5 | 11.3±1.3 | 7.4±1.8 |
| 5 (25 d) | 14.2±1.8 | 11.0±0.4 | 11.5±0.7 | 10.7±0.7 | 9.5±1.3 | 10.5±0.7 | 11.3±0.2 | 9.6±0.5 |
| 6 (30 d) | 11.8±1.7 | 10.3±0.5 | 11.9±0.8 | 10.6±0.7 | 9.5±0.5 | 9.7±0.5 | 9.5±1.6 | 8.2±1.1 |
| 7 (35 d) | 10.8±1.5 | 8.7±1.3 | 12.4±0.6 | 11.5±0.8 | 10.0±0.8 | 10.0±1.8 | 9.4±0.8 | 8.3±0.9 |
| 8 (40 d) | 10.3±1.0 | 8.3±0.6 | 11.5±0.7 | 9.7±0.7 | 8.7±1.3 | 8.9±1.1 | 8.3±0.6 | 6.5±0.5 |
| 9 (45 d) | 10.3±1.2 | 8.3±0.8 | 11.0±0.4 | 9.9±0.4 | 7.6±0.6 | 7.5±0.7 | 9.0±1.1 | 7.6±1.4 |
| 10 (50 d) | 9.7±1.2 | 7.2±0.4 | 10.5±0.4 | 9.1±0.3 | 6.3±0.5 | 6.3±0.4 | 10.4±0.7 | 8.0±1.7 |
| 11 (55 d) | 9.5±0.7 | 8.0±0.7 | 9.0±0.3 | 8.0±0.4 | 6.0±0.3 | 5.8±0.5 | 10.8±0.8 | 8.5±0.6 |
| 12 (60 d) | 9.0±0.3 | 7.5±0.5 | 8.0±0.5 | 6.8±0.6 | 5.8±0.4 | 5.5±0.4 | 8.4±1.4 | 6.6±0.9 |

c) Length and diameter of the inner, middle and outer leaves collected from 5-month-old greenhouse grown cabbage plants grown in a standard growth chamber conditions in Experiment 2

| Leaf type | **BN4383** | | **BN4384** | | **BN4071** | | **BN4161** | |
| --- | --- | --- | --- | --- | --- | --- | --- | --- |
|  | Length (cm) | Diameter (cm) | Length (cm) | Diameter (cm) | Length (cm) | Diameter (cm) | Length (cm) | Diameter (cm) |
| Inner | 14.1±1.2 | 15.6±1.6 | 12.8±1.8 | 18.5±3.2 | 11.8±0.9 | 12.2±1.6 | 13.1±1.2 | 14.3±3.1 |
| Middle | 18.2±1.8 | 22.2±2.2 | 15.5±2.0 | 23.6±3.2 | 15.3±2.0 | 17.9±1.7 | 16.3±1.3 | 24.0±2.5 |
| Outer | 24.8±4.0 | 28.3±3.6 | 25.6±4.2 | 31.2±3.0 | 22.9±2.2 | 24.6±2.8 | 20.8±2.1 | 28.5±4.5 |

**Table S2.** Cuticular wax load (% of total wax) in two high-wax lines, BN4383 and BN4384, and two low-wax lines, BN4071 and BN4161, at the leaf position 4 of 10-week-old plants.

| Type | Compound | Molecular formulae | % of total wax | | | |
| --- | --- | --- | --- | --- | --- | --- |
|  |  |  | BN4383 | BN4384 | BN4071 | BN4161 |
| Alkanes | Heptacosane | C_27_H_56_ | 0.15 | 0.12 | 0.14 | 0.09 |
|  | Nonacosane | C_29_H_60_ | 30.55 | 32.14 | 26.84 | 31.19 |
|  | Triacontane | C_30_H_62_ | 0.59 | 0.67 | 0.84 | 0.92 |
|  | N-hentriacosane | C_31_H_64_ | 3.45 | 2.32 | 5.80 | 2.20 |
| Primary  alcohols | 1-Tetracosanol | C_24_H_50_O | 0.07 | 0.03 | 0.06 | 0.03 |
|  | 1-Hexacosanol | C_26_H_54_O | 1.69 | 1.04 | 1.41 | 0.90 |
|  | 1-Heptacosanol | C_27_H_56_O | 2.72 | 4.15 | 2.79 | 4.27 |
|  | 1-Octacosanol | C_28_H_58_O | 0.73 | 0.58 | 0.71 | 0.62 |
|  | 1-Nonacosanol | C_29_H_60_ O | 1.18 | 0.92 | 1.16 | 1.13 |
| Aldehydes | Octacosanal | C_28_H_56_O | 0.67 | 0.46 | 0.42 | 0.36 |
|  | Triacontana | C_30_H_60_O | 5.88 | 5.59 | 4.75 | 6.60 |
| Secondary alcohols | 14,15-Nonacosanol | C_29_H_61_O_2_ | 13.71 | 13.17 | 15.09 | 13.98 |
| Ketol | 15 (or 16)-Hydroxynonacosan-14-one | C_29_H_58_O_2_ | 1.90 | 1.61 | 2.18 | 1.94 |
| Ketones | 15-Nonacosanone | C_29_H_58_O | 31.33 | 31.22 | 31.89 | 29.58 |
| Unidentified |  |  | 5.39 | 5.98 | 5.90 | 6.18 |
| Total |  |  | 100 | 100 | 100 | 100 |

**Table S3.** Probability values (*p* value) indicating statistically significant difference among four cabbage inbred lines in wax composition.

| **Compound name** | **Carbon number** | **P value** | |
| --- | --- | --- | --- |
|  |  | **Genotype (µg/cm^2^)** | **Genotype (Mol %)** |
| Total wax |  | <0.001 | <0.001 |
| Alkanes | C_27_H_56_ | <0.001 | <0.001 |
|  | C_29_H_60_ | <0.001 | <0.001 |
|  | C_30_H_62_ | <0.001 | <0.001 |
|  | C_31_H_64_ | <0.001 | <0.001 |
| Primary alcohols | C_24_H_50_O | <0.001 | 0.001 |
|  | C_26_H_54_O | <0.001 | <0.001 |
|  | C_27_H_56_O | <0.001 | <0.001 |
|  | C_28_H_58_O | <0.001 | 0.007 |
|  | C_29_H_60_ O | 0.001 | 0.109 |
| Aldehydes | C_29_H_61_O_2_ | <0.001 | <0.001 |
|  | C_30_H_63_O_2_ | <0.001 | 0.008 |
| Secondary alcohols | C_29_H_61_O_2_ | <0.001 | 0.062 |
| Ketol | C_29_H_58_O_2_ | 0.001 | <0.001 |
| Ketones | C_29_H_58_O | <0.001 | <0.001 |

**Table S4.** Probability values (*p* value) indicating statistical significance of differences among different leaf samples for each cabbage line.

| **Gene name** | **Accession Number** | **First-formed leaves at three sampling points** | | | |
| --- | --- | --- | --- | --- | --- |
|  |  | **BN4383** | **BN4384** | **BN4071** | **BN4161** |
| *BoLACS1.1* | Bol002529 | 0.418 | 0.433 | <0.001 | 0.136 |
| *BoLACS1.3* | Bol029614 | 0.180 | 0.268 | 0.033 | 0.098 |
| *BoLACS1.4* | Bol002590 | 0.003 | 0.104 | 0.012 | 0.005 |
| *BoKCS1.1* | Bol018447 | 0.688 | 0.035 | 0.001 | 0.116 |
| *BoKCS1.2* | Bol000521 | 0.256 | 0.004 | 0.016 | 0.001 |
| *BoKCS1.3* | Bol040715 | 0.061 | 0.022 | 0.005 | 0.060 |
| *BoKCR1.1* | Bol010474 | 0.002 | 0.411 | 0.064 | 0.093 |
| *BoECR* | Bol044348 | 0.000 | 0.317 | 0.065 | 0.006 |
| *BoCER3.3* | Bol012187 | 0.045 | 0.410 | 0.804 | 0.139 |
| *BoCER3.4* | Bol015584 | 0.869 | 0.008 | 0.003 | 0.001 |
| *BoMAH1.1* | Bol016302 | 0.057 | 0.488 | 0.009 | 0.036 |
| *BoLTP2.1* | Bol017820 | 0.179 | 0.468 | 0.007 | <0.001 |
| *BoLTP2.2* | Bol025301 | 0.007 | 0.881 | <0.001 | 0.001 |
| *BoLTP2.3* | Bol025304 | 0.004 | 0.131 | 0.011 | <0.001 |
| *BoFAR3.1* | Bol013612 | <0.001 | 0.048 | 0.002 | 0.011 |
| *BoFAR3.2* | Bol017561 | 0.015 | <0.001 | 0.039 | 0.003 |
| *BoWSD1.2* | Bol020399 | <0.001 | 0.031 | 0.005 | 0.013 |
| *BoWSD1.3* | Bol024738 | 0.008 | 0.208 | 0.069 | 0.001 |
| *BoABCG11.1* | Bol013247 | 0.012 | 0.702 | <0.001 | 0.010 |
| *BoABCG11.2* | Bol030816 | 0.382 | 0.181 | 0.129 | 0.001 |
| Six different leaf positions | | | | | |
| *BoLACS1.4* | Bol002590 | <0.001 | <0.001 | <0.001 | 0.004 |
| *BoKCS1.1* | Bol018447 | <0.001 | <0.001 | 0.005 | 0.002 |
| *BoKCR1.1* | Bol010474 | <0.001 | <0.001 | <0.001 | 0.001 |
| *BoCER3.3* | Bol012187 | 0.004 | <0.001 | 0.001 | <0.001 |
| *BoCER3.4* | Bol015584 | <0.001 | 0.001 | <0.001 | <0.001 |
| *BoMAH1.1* | Bol016302 | <0.001 | <0.001 | 0.001 | <0.001 |
| *BoLTP2.1* | Bol017820 | 0.008 | <0.001 | <0.001 | <0.001 |
| *BoLTP2.2* | Bol025301 | <0.001 | 0.001 | 0.001 | <0.001 |
| *BoLTP2.3* | Bol025304 | <0.001 | 0.007 | <0.001 | <0.001 |
| Outer, middle and inner leaf from a cabbage head | | | | | |
| *BoLACS1.4* | Bol002590 | <0.001 | 0.020 | <0.001 | <0.001 |
| *BoKCS1.1* | Bol018447 | 0.001 | 0.008 | 0.020 | <0.001 |
| *BoKCR1.1* | Bol010474 | 0.005 | 0.012 | 0.237 | 0.002 |
| *BoCER3.3* | Bol012187 | 0.027 | 0.072 | 0.037 | 0.175 |
| *BoCER3.4* | Bol015584 | 0.018 | 0.008 | 0.018 | 0.484 |
| *BoMAH1.1* | Bol016302 | 0.109 | 0.006 | 0.061 | <0.001 |
| *BoLTP2.1* | Bol017820 | <0.001 | 0.008 | 0.000 | <0.001 |
| *BoLTP2.2* | Bol025301 | 0.012 | 0.002 | 0.018 | <0.001 |
| *BoLTP2.3* | Bol025304 | <0.001 | 0.022 | 0.168 | <0.001 |

**Table S5.** Probability values (*p* value) indicating statistical significance of differences among different leaf samples, genotypes and genotype x leaf samples in three different experiments.

| **Gene name** | **Accession Number** | **Sampling points**  **(Exp 1)** | **Genotype** | **Genotype × Sampling points** |
| --- | --- | --- | --- | --- |
| *BoLACS1.1* | Bol002529 | 0.229 | <0.001 | 0.918 |
| *BoLACS1.3* | Bol029614 | 0.009 | 0.008 | 0.566 |
| *BoLACS1.4* | Bol002590 | <0.001 | 0.001 | 0.102 |
| *BoKCS1.1* | Bol018447 | 0.185 | 0.282 | 0.235 |
| *BoKCS1.2* | Bol000521 | <0.001 | 0.001 | <0.001 |
| *BoKCS1.3* | Bol040715 | <0.001 | <0.001 | <0.001 |
| *BoKCR1.1* | Bol010474 | 0.001 | 0.462 | 0.415 |
| *BoECR* | Bol044348 | <0.001 | 0.004 | 0.002 |
| *BoCER3.3* | Bol012187 | 0.684 | 0.001 | 0.042 |
| *BoCER3.4* | Bol015584 | 0.215 | 0.003 | 0.046 |
| *BoMAH1.1* | Bol016302 | 0.335 | <0.001 | 0.003 |
| *BoLTP2.1* | Bol017820 | <0.001 | <0.001 | <0.001 |
| *BoLTP2.2* | Bol025301 | <0.001 | <0.001 | 0.010 |
| *BoLTP2.3* | Bol025304 | <0.001 | <0.001 | <0.001 |
| *BoFAR3.1* | Bol013612 | <0.001 | <0.001 | <0.001 |
| *BoFAR3.2* | Bol017561 | <0.001 | 0.001 | 0.013 |
| *BoWSD1.2* | Bol020399 | <0.001 | <0.001 | <0.001 |
| *BoWSD1.3* | Bol024738 | <0.001 | 0.021 | 0.004 |
| *BoABCG11.1* | Bol013247 | 0.002 | 0.002 | 0.011 |
| *BoABCG11.2* | Bol030816 | <0.001 | <0.001 | 0.014 |
| **Gene name** | **Accession Number** | **Leaf positions**  **(Exp 2)** | **Genotype** | **Genotype × leaf positions** |
| *BoLACS1.4* | Bol002590 | <0.001 | <0.001 | 0.478 |
| *BoKCS1.1* | Bol018447 | <0.001 | 0.008 | <0.001 |
| *BoKCR1.1* | Bol010474 | <0.001 | <0.001 | <0.001 |
| *BoCER3.3* | Bol012187 | <0.001 | <0.001 | <0.001 |
| *BoCER3.4* | Bol015584 | <0.001 | <0.001 | <0.001 |
| *BoMAH1.1* | Bol016302 | <0.001 | <0.001 | <0.001 |
| *BoLTP2.1* | Bol017820 | <0.001 | <0.001 | <0.001 |
| *BoLTP2.2* | Bol025301 | <0.001 | <0.001 | <0.001 |
| *BoLTP2.3* | Bol025304 | <0.001 | <0.001 | <0.001 |
| **Gene name** | **Accession Number** | **Leaf type**  **(Exp 3)** | **Genotype** | **Genotype × Leaf type** |
| *BoLACS1.4* | Bol002590 | <0.001 | <0.001 | <0.001 |
| *BoKCS1.1* | Bol018447 | <0.001 | <0.001 | <0.001 |
| *BoKCR1.1* | Bol010474 | 0.042 | 0.001 | 0.001 |
| *BoCER3.3* | Bol012187 | 0.157 | 0.532 | 0.001 |
| *BoCER3.4* | Bol015584 | <0.001 | <0.001 | <0.001 |
| *BoMAH1.1* | Bol016302 | <0.001 | <0.001 | <0.001 |
| *BoLTP2.1* | Bol017820 | <0.001 | <0.001 | <0.001 |
| *BoLTP2.2* | Bol025301 | <0.001 | <0.001 | <0.001 |
| *BoLTP2.3* | Bol025304 | <0.001 | <0.001 | <0.001 |
